# Biobehavioral Interactions between Endocannabinoid and Hypothalamic-pituitary-adrenal Systems in Psychosis: A Systematic Review

**DOI:** 10.2174/1570159X21666230801150032

**Published:** 2023-08-04

**Authors:** Marco Colizzi, Riccardo Bortoletto, Giulia Antolini, Sagnik Bhattacharyya, Matteo Balestrieri, Marco Solmi

**Affiliations:** 1 Unit of Psychiatry, Department of Medicine (DAME), University of Udine, Udine 33100, Italy;; 2 Department of Psychosis Studies, Institute of Psychiatry, Psychology and Neuroscience, King’s College London, London SE5 8AF, UK;; 3 Child and Adolescent Neuropsychiatry Unit, Maternal-Child Integrated Care Department, Integrated University Hospital of Verona, Verona 37126, Italy;; 4 Department of Psychiatry, University of Ottawa, Ottawa, ON, Canada;; 5 Department of Mental Health, The Ottawa Hospital, Ottawa, ON, Canada;; 6 Department of Child and Adolescent Psychiatry, Charité Universitätsmedizin, Berlin, Germany

**Keywords:** Multisystem disorder, central nervous system, mental health, co-morbidity, environment, organ systems, body systems, brain

## Abstract

**Background:**

The diathesis-stress paradigm and the cannabinoid-hypothesis have been proposed as possible pathophysiological models of schizophrenia. However, they have historically been studied independently of each other.

**Objective:**

This PRISMA 2020-compliant systematic review aimed at reappraising the interplay between the hypothalamic-pituitary-adrenal (HPA) axis and the endocannabinoid (eCB) system in psychosis-spectrum disorder risk and outcome.

**Methods:**

All pathophysiological and outcome clinical studies, concomitantly evaluating the two systems in psychosis-spectrum disorder risk and different stages of illness, were gathered from electronic databases (Pubmed, Web of Science, and Scopus), and discussed.

**Results:**

41 eligible outputs were extracted, focusing on at least a biological measure (9 HPA-related studies: 4 eCB-interventional, 1 HPA-interventional, 1 both HPA-interventional and non-interventional, 3 non-interventional; 2 eCB-related studies: non-interventional), environmental measures only (29 studies: 1 eCB- interventional, 28 non-interventional), and genetic measures (1 study: non-interventional). Independent contributions of aberrancies in the two systems to the physiopathology and outcome of psychosis were confirmed. Also, concomitant alterations in the two systems, either genetically defined (*e.g*., CNR1 genetic variation), biologically determined (*e.g*., dysfunctional HPA axis or endocannabinoid signaling), or behaviorally imputed (*e.g*., cannabis use, stress exposure, and response), were consistently reported in psychosis. Further, a complex biobehavioral perturbation was revealed not only within each system (*e.g*., cannabis use affecting the eCB tone, stress exposure affecting the HPA axis), but also across the two systems (*e.g*., THC affecting the HPA axis, childhood trauma affecting the endocannabinoid signaling).

**Conclusion:**

There is a need to concomitantly study the two systems’ mechanistic contribution to psychosis in order to establish more refined biological relevance.

## 1 INTRODUCTION

In recent years, attempts have been made at integrating the different proposed pathophysiological models of schizophrenia into a developmental process whereby a sequence of insults to the central nervous system would make individuals more susceptible to any potential environment-induced neurophysiological perturbation [1-3]. Such a conceptual model tries to account for different clinical phenomena occurring in the context of the disorder [4], such as being sensitive, along with numerous other risk factor exposures, to the psychosis-inducing effects of stress [5] or cannabis use [6]. Accumulating evidence regarding stress-related as well as cannabis-associated psychosis has led to the formulation of two different psychosis models that have historically been studied independently of each other [7, 8].

The 60-year old diathesis-stress model of schizophrenia hypothesizes an interaction between psychosocial stress exposure and genetic susceptibility in the etiopathogenesis of psychosis [7]. Accordingly, in the presence of a preexisting vulnerability, stress would trigger the onset of an acute psychotic episode [9]. Subsequent refinements have encompassed the neurobiological underpinnings of the stress response, providing evidence for the role of the hypothalamic-pituitary-adrenal (HPA) axis activation in triggering a cascade of neurochemical events that can elicit a psychotic reaction [10, 11]. More recent models propose that the HPA axis is widely involved in structural and functional brain networks at the neurodevelopmental, epigenetic, neurotransmitter, and inflammatory levels, with stress leading to dopamine dysregulation, on par with other risk factors for psychosis [12]. While HPA axis abnormalities are reported across the psychosis spectrum, less clear is whether they are entirely accounted for by psychosocial stressors [13], calling into question whether the association between stressful life events and psychosis relapse might be mediated by the HPA axis.

Following anecdotical evidence that cannabis use may induce paranoid reactions and acute psychotic symptoms [14], the last five decades have seen a number of systematic investigations consistently indicating an association between cannabis use and psychosis [6, 15], thus fueling a cannabinoid-hypothesis of psychosis. However, the causal interpretation of such an association has been questioned [16], urging the assessment of alternative explanations, such as the confounding effect of other substance use, preexisting higher psychopathology in cannabis users, self-medication (reverse causality), and shared genetic vulnerability. A recent reappraisal of the topic according to Bradford-Hill criteria points in the direction of a robust cause-effect relationship, though of modest strength, with the risk of psychosis increasing as a function of cannabis frequency and potency as well as specific genetic or neurophysiological background [17-19]. Independent of such evidence, a more recent systematic review and meta-analysis indicates higher blood and cerebrospinal fluid (CSF) endocannabinoid levels as well as higher cannabinoid receptor type 1 (CB1) expression on peripheral immune cells in psychosis patients as compared to healthy controls [20]. Also, such parameters were found to be sensitive to symptom severity, stage of illness, and response to treatment [20]. While it is unclear whether eCB alterations are causally linked to psychosis or a consequence of the disease process, the evidence raises the possibility that such aberrancies in the eCB system might represent a biomarker of psychosis status and outcome.

Accumulating evidence suggests that the two psychosis models are not mutually exclusive, as indicated by evidence of simultaneous and interconnected alterations in the HPA axis and eCB systems in psychosis [8]. In particular, the role of the eCB system in mediating the HPA axis response to stress has been hypothesized, with implications for the development of psychosis [21]. Also, such biobehavioral interactions have been suggested to be relevant for both the premorbid phase and the psychosis progression [12]. Thus, the concomitant assessment of both systems may be a promising strategy to further advance our knowledge of schizophrenia-spectrum disorders. Within this systematic review, we have tried to better clarify the interplay between the HPA axis and eCB system in psychosis, by gathering and discussing all available clinical data, including both physiopathological and outcome studies.

## 2 MATERIALS AND METHODS

### 2.1 Inclusion and Exclusion Criteria

All clinical evidence regarding the interplay between the two systems in psychosis risk and outcome was systematically brought together, defining inclusion criteria as outlined: (1) cross-sectional studies, case-control studies, cohort studies, case-reports/series, and randomized controlled trials; (2) studies exploring HPA axis- and eCB system-related biological correlates (*e.g*., cortisol salivary or plasma levels, eCBs/acylethanolamines (AEs) plasma levels) in relation to psychosis clinical high-risk (CHR) state or psychosis different stages of illness, compared or not to healthy individuals; (3) studies exploring HPA axis- and eCB system-related behavioral correlates (*e.g*., experimental stress exposure or lifetime stressful events, experimental cannabinoid exposure or lifetime cannabis use) in relation to psychosis CHR state or psychosis different stages of illness, compared or not to healthy individuals, with or without biological correlates. Exclusion criteria were as follows: (1) narrative reviews, systematic reviews, meta-analyses, and animal studies; (2) studies investigating HPA axis and eCB system interplay but independently of psychosis risk, pathophysiology, and outcomes; (3) studies investigating CHR state or psychosis at different stages of illness as per the eCB system modulation but not the HPA axis; (4) studies investigating CHR state or psychosis at different stages of illness as per the HPA axis modulation but not the eCB system.

### 2.2 Search Strategy and Data Extraction

**Fig. (1):**
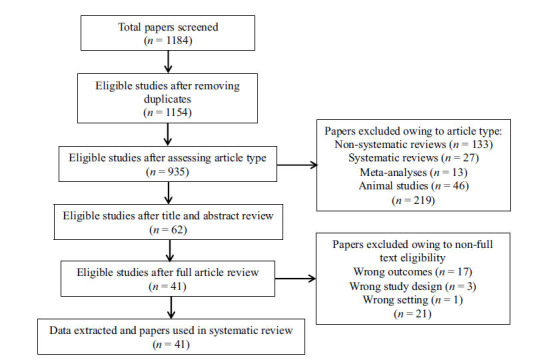
PRISMA flow chart.

A literature search was conducted using electronic databases (Pubmed, Web of Science, and Scopus) for any published original study written in English, using a combination of broad-meaning search terms describing and/or concerning the HPA axis (‘cortisol’, ‘HPA’, ‘hypothalamus’, ‘pituitary’, ‘adrenal’, ‘axis’, ‘stress’, ‘trauma’, and ‘adversity’), the eCB system (‘cannab*’, ‘CBD’, ‘THC’, ‘delta-9-tetrahydrocanna-binol’, ‘Δ-9-tetrahydrocannabinol’, ‘marij*’, ‘marih*’, ‘sativa’, and ‘indica’), and psychosis (‘FEP’, ‘first-episode’, ‘schizophrenia’, ‘psychosis’, ‘UHR’, and ‘CHR’), on 30 November, 2022. Reference lists of eligible studies were screened to identify additional eligible research. Data screening and extraction were conducted according to a two-step selection process (conventional double-screening), performed by two researchers independently from each other (R.B. and G.A.). In the instances of conflicting opinions regarding papers' inclusion, a consensus was reached through discussion with a third senior reviewer (M.C.). The entire process was translated into a PRISMA 2020-compliant systematic review flow diagram (Fig. **1**) [22].

### 2.3 Risk of Bias Assessment

Considering the methodological heterogeneity of studies included in this review (Table **1**), a risk of bias assessment was conducted, as previously done [23, 24]. An adapted set of criteria suggested by the Agency for Healthcare Research and Quality (AHRQ) guidance was deemed as appropriate to perform a quality of studies assessment [25]. Similarities and differences between selected papers were appraised by extracting information about study characteristics, including study design, study population (*e.g*., healthy subjects, schizophrenia patients, individuals at clinical high-risk for psychosis), gender, age, HPA axis/stress measure (*e.g*., cortisol salivary or plasma levels, childhood trauma), adequate HPA axis/stress evaluation (*e.g*., single or multiple assessment), eCB system measure (*e.g*., cannabinoid dosage and administration route, eCB assessment in tissues, cannabis use), and adequate eCB system evaluation (*e.g*. daily administration, single or multiple assessment) (Table **3**). Besides, the risk of systematic bias across all studies was ruled out by screening all papers for potentially confounding variables (*e.g*., age, gender, education, tobacco use) (Table **3**).

The full study protocol is available at https://doi.org/10.17605/OSF.IO/ERQPG.

## 3 RESULTS

### 3.1 Identified Studies for Inclusion in the Systematic Review

In summary, 1184 records were identified through the initial data search. After excluding duplicates as well as articles owing to article type (systematic and non‐systematic reviews), by using a three‐step screening approach, titles, abstracts, or full texts of all records were screened against the inclusion and exclusion criteria (Fig. **1**). A final list of 41 studies was used for systematic analysis in this review, investigating different aspects of the interplay between the hypothalamic-pituitary-adrenocortical (HPA) axis and endocannabinoid (eCB) system modulation in psychosis risk, physiopathology, and outcomes (Table **1**). These included (i) the effect of exogenous cannabinoid administration on HPA axis functioning in healthy subjects, CHR individuals, and psychosis patients at different stages of illness (4 studies; Table **1**); (ii) the effect of cannabis use on HPA axis functioning in healthy subjects, CHR individuals, and psychosis patients at different stages of illness (3 studies; Table **1**); (iii) the effect of cannabis use and stress exposure on HPA axis functioning and dopamine system in CHR individuals and psychosis patients at different stages of illness (3 studies; Table **1**); (iv) the effect of childhood trauma and exogenous cannabinoid administration over psychosis risk in healthy subjects (1 study; Table **1**); (v) the effect of childhood trauma and cannabis use over psychosis risk in the general population and CHR individuals or psychosis exacerbation in patients at different stages of illness (28 studies; Table **1**); (vi) the effect of childhood trauma on eCB/acylethanolamine (AE) and its precursor levels in cannabis-using and non-using CHR individuals and psychosis patients (2 studies; Table **1**); and (vii) the effect of genetic variation in cannabinoid receptor 1 (CNR1) and cannabis use over perceived stress and neurocognition in patients at their first episode of psychosis (FEP) (1 study; Table **1**). A detailed presentation of the results is reported in Table **2**. Additional data on the methodological quality of studies included in the review are reported in Table **3**. A brief synthesis of the main results is presented below.

### 3.2 Effect of Exogenous Cannabinoid Administration on HPA Axis Functioning in Healthy Subjects, CHR Individuals, and Psychosis Patients at Different Stages of Illness

This review identified four challenge studies addressing the effect of exogenous cannabinoid administration on HPA axis functioning (Tables **1** and **2**), using similar but not overlapping methodologies (Table **3**) in terms of the study population (schizophrenia/schizoaffective disorder [26], clinical high-risk (CHR) individuals [27, 28], and healthy subjects [26-29]), sample size (range: 16-58 subjects), cannabinoid exposure (delta-9-tetrahydrocannabinol (THC) [26, 29], cannabidiol (CBD) [27, 28]), cannabinoid dosage (2.5/5 mg [26] and 1.19 mg/2 ml [29] for THC; 600 mg for CBD [27, 28]), cannabinoid mode of administration (intravenous for THC [26, 29], oral for CBD [27, 28]), cannabinoid period of exposure (single administration [29], 3 days [26], 7 days [27, 28]), and outcome measure (serum cortisol [26-29], serum prolactin [26]). All studies adopted a placebo-controlled design and were sufficiently rigorous in terms of subjects’ comparability and/or excluding/adjusting for confounding factors (Table **3**).

Challenge studies have indicated THC administration to disrupt the HPA axis, as indexed by higher cortisol and prolactin levels [26] as well as flattened diurnal cortisol decrease [29], when compared to the placebo condition. Independent of the THC challenge, intrinsic differences in the HPA axis between schizophrenia patients and healthy controls were reported. Instead, the THC-induced HPA axis disruption did not seem to change as a function of the diagnosis status, with non-significantly different patterns across the two groups [26]. However, among healthy subjects, THC was found to result in greater cortisol disruption over time in individuals presenting with THC-induced psychosis-like experiences (PLE) when compared to those who did not develop PLE under the effect of the drug [29].

Another line of research investigated the potential therapeutic effect of CBD in CHR individuals through the modulation of the HPA axis and stress response. A first study indicated blunted cortisol response as well as greater anxiety and experience of public speaking stress following experimental stress in CHR individuals under placebo when compared to healthy controls, with CBD administration partially reversing such aberrant biobehavioral responses in CHR individuals [27]. A further study from the same group found that cortisol reactivity following experimental stress negatively correlated with the right parahippocampal activation during fear processing in healthy controls and that such association differentiated the diagnosis status, being statistically different between healthy subjects and CHR individuals. Finally, the absent pattern of coupling between neural response to fear and cortisol response to stress in CHR individuals was not recovered/normalized by CBD administration [28].

### 3.3 Effect of Cannabis use on HPA Axis Functioning in Healthy Subjects, CHR Individuals, and Psychosis Patients at Different Stages of Illness

In total, three studies evaluated whether cannabis-using and non-using psychosis patients at different stages of illness differ in terms of HPA axis functioning (Tables **1** and **2**). The review identified similar but not overlapping methodologies (Table **3**) in terms of the study population (schizophrenia [30], recent-onset psychosis (ROP) patients [31], CHR individuals [32], and healthy subjects [30-32]), sample size (range: 43-103 subjects), cannabinoid exposure (self-reported cannabis use [30-32] and/or cannabis urine screen [32]), cannabinoid period of exposure (before the onset of psychosis [30], last week/last month [32], current [31]), and outcome measure (salivary cortisol [30-32] and cortisol suppression ratio after dexamethasone (DSTR; [31]). The first of these studies found that, as compared to healthy controls, cannabis-using schizophrenia patients have higher morning cortisol levels and a flattened cortisol awakening response (CAR), while non-using patients would present with a cortisol pattern like that observed among healthy controls [30]. Another study also found higher cortisol levels among CHR individuals, as compared to healthy controls, with cannabis-using patients presenting with furtherly increased cortisol levels when compared to non-using patients [32]. Finally, a more recent study confirmed a flattened diurnal cortisol slope as a function of CU, but independent of ROP diagnosis [31].

### 3.4 Effect of Cannabis use and Stress Exposure on HPA Axis Functioning and Dopamine System in CHR Individuals and Psychosis Patients at Different Stages of Illness

Three studies investigated the effect of CU on the HPA axis functioning in patients with psychosis at different stages of illness experimentally exposed to stress [33, 34] or with a personal history of stressful life events (SLEs) and childhood trauma (CT) [31] (Tables **1** - **2**). Two of them further explored the interaction between CU, stress, and dopamine signaling [33, 34]. Using multiple linear regression analysis to study the association between exposure to environmental factors and HPA axis measures in ROP patients, one study found a role for cannabis in disrupting the diurnal cortisol slope, but not SLEs or CT [31]. Other studies revealed decreased stress-induced cortisol response [33] and dopamine release in the prefrontal cortex [33] and striatum [34] of cannabis-using CHR individuals, as compared to non-using patients. Stress-induced prefrontal dopamine release correlated positively with the stress-induced cortisol response but negatively with the stress-induced change in attenuated psychotic symptoms [33]. Finally, stress-induced striatal dopamine reactivity was also found to correlate with stress-induced cortisol response, but only in non-using CHR individuals, suggesting a possible decoupling of HPA response from dopaminergic signaling in the context of CU [34].

### 3.5 Effect of Childhood Trauma and Exogenous Cannabinoid Administration Over Psychosis Risk in Healthy Subjects

This review identified a single study specifically investigating whether childhood trauma (CT) increases the risk of presenting with PLE under the acute effects of THC in otherwise healthy cannabis-using subjects (Tables **1**-**3**). CT was associated with PLE during THC intoxication, with cognitive fusion, *i.e*., the inability to defuse from internal experience, accounting for increased THC-induced PLE among those with CT. Interestingly, CT was also found to be associated with schizotypy, independent of the acute THC challenge [35].

### 3.6 Effect of Childhood Trauma and/or SLEs and Cannabis use Over Psychosis Risk in the General Population and CHR Individuals or Psychosis Exacerbation in Patients at Different Stages of Illness

Out of 41 studies, 28 of them investigated the interplay between childhood trauma (CT) and cannabis use (CU) in conferring psychosis risk or exacerbating psychosis symptoms among both healthy individuals and patients (Tables **1** and **2**). The review identified similar but not overlapping methodologies (Table **3**) in terms of study population (schizophrenia/schizoaffective disorder [36-39], bipolar affective disorder [37, 39, 40], ROP patients [41, 42], FEP patients [37, 43], CHR individuals [44], general population [45-59], healthy subjects [43, 60-62], cannabis-using subjects [37, 63]), sample size (range: 112-7403 subjects), type of CT (sexual [36-38, 40-57, 59-63], physical/violence [36, 38, 41-44, 46, 47, 49, 50, 53-56, 60-63], emotional [36, 38, 41, 42, 44, 47, 49, 50, 53, 56, 57, 60-63], domestic violence [46], peer victimization/bullyism [56, 57]), cannabinoid exposure (use [37-63], disorder/dependence [36, 40]), cannabinoid period of exposure (lifetime [36-40, 42-56, 58-60, 62], past 12 months [54, 57, 61], past 6 months [41], past month [63], current [44, 56, 61], at follow-up assessment [55, 58]), and outcome measure (Composite International Diagnostic Interview, CIDI [45, 47-50, 53, 56, 58], Structured Clinical Interview for Disorders, SCID [36, 42, 45, 53, 58], Schedule for Assessment in Neuropsychiatry, SCAN [38, 51, 52], Schedule for Affective Disorders and Schizophrenia for School-Age Children, K-SADS [46], Diagnostic Interview for Genetic Study, DIGS [40], computerized method for diagnoses of functional psychoses, DIAX [49, 50], Operational Criteria System, OPCRIT checklist [41, 43], Community Assessment of Psychic Experiences, CAPE [47, 60, 61, 63], Psychosis Screening Questionnaire, PSQ [51, 52, 54], The Content of Attenuated Positive Symptoms, CAPS [44], Prodromal Questionnaire, PQ-16 [57], Prodromal Questionnaire-Brief Version, PQB [37], Positive and Negative Symptoms Scale, PANSS [36, 39, 42], Paranoia Scale, PS [37], Self-Report Symptom Checklist-90-R, SCL-90-R [50, 55], Columbia-Suicide Severity Rating Scale, C-SSRS [36], Global Assessment of Functioning Scale, GAF [36], Medication Adherence Rating Scale, MARS [36], Shortened Quality of Life questionnaire, S-QoL-18 [36], Wechsler Intelligence Scale for Children [56], The Brief Assessment of Cognition in Schizophrenia, BACS [39], and white matter integrity [62]).

Among studies investigating the independent effect of CT on psychosis risk, evidence tips the scale towards a deleterious effect of CT in terms of increasing the risk of getting a psychosis diagnosis and experiencing psychotic symptoms [38, 42, 44, 46-48, 53, 54, 57, 61, 63] among different study samples, including schizophrenia/schizoaffective disorder [38] and ROP patients [42], CHR individuals [44], general population [46-48, 53, 54, 57], healthy subjects [61], and cannabis-using subjects [63]. Also, CT has been found to anticipate psychosis onset [39] and predict a poorer clinical course [36] among patient samples. Single studies suggest that such association may be gender-specific, being observed in female individuals only [59], and only true for those exposed to high levels of CT [41]. Further, other evidence supports an association between SLEs and higher risk of incident clinical psychosis [58]. However, a few studies have not suggested any association between CT and psychosis-related measures [40, 43, 45, 51, 60].

Of those outputs focusing on the main effect of CU, most found that it increases the risk of developing psychosis and presenting with psychotic symptoms [36, 40, 42, 46, 47, 53, 57, 58, 61, 63], possibly because of recent/current use [44, 54] as well as regular and more severe use [38, 41]. Also, another study suggested an earlier psychosis onset as a function of CU [39]. However, a few studies did not suggest any association between CU and psychosis-related measures [43, 45, 48, 51, 60].

More importantly, some studies converged in suggesting an interaction effect between CT and CU over the risk of PLE [46, 48, 61], psychotic symptoms in bipolar disorder [40], and psychosis diagnosis [51], possibly because of earlier CT [47] as well as early [45], recent [54], and regular [38] CU. Mediating and moderating effects of either CT or CU were reported in the association between any of the two variables and both PLE [53, 63] and psychosis diagnosis [53], with also relevance for psychosis age of onset [39]. Further, it is worth mentioning that the history of SLEs itself was found to interact with CU in increasing the risk of incident clinical psychosis [58]. However, a few studies did not suggest an interaction between CT and CU in either increasing psychosis risk [42, 44, 49, 59-61] or affecting functional and clinical outcomes of psychosis, apart from prolonging the duration of hospitalization [36], possibly because of a confounding effect of stimulants [52] and CU frequency and potency [43].

Finally, some studies indicated that the interaction effect between CU and CT on PLE [37, 50, 55-57, 60, 61] and ROP [41] is further complexified by additional factors that would have a role in conferring a greater risk, including catechol-O-methyltransferase (COMT) gene [60, 61], urbanicity [50, 55, 56], SLEs [41], threat-induced cognitive biases [57], cognitive alteration [56], and cognitive fusion [37]. Also, the interaction between CT and CU was found to be relevant for the occurrence of PLE in the context of affective disorders [55], along with affective and negative symptoms [56]. Further, white matter integrity, as an intermediate phenotype of psychosis, was reported to be disrupted in otherwise healthy subjects with a history of both CT and CU [62].

### 3.7 Effect of Childhood Trauma or Stress on eCB/AE and their Precursor Levels in Cannabis-using and Non-using CHR Individuals and Psychosis Patients

This review found two studies to focus on the association between stress and abnormalities in the eCB signaling, including precursors and endogenous lipids belonging to the AE fatty acid (FA) amide family, also as a function of cannabis use (Tables **1**-**3**). The first research on this topic suggested specific alterations, mainly in schizophrenia patients with a former history of cannabis use, with arachidonic acid (AA) and total 16- and 18-carbon monounsaturated and saturated FAs (16, 18 m + sFAs) being downregulated as well as linoleic acid being upregulated in response to stress. Instead, among cannabis-naïve patients, a total of 16,18m + sFAs were increased as a function of stress while AA and LA were not affected [64]. A later study suggested higher palmitoylethanolamide (PEA) and anandamide (AEA) levels among CHR individuals exposed to CT, as compared to healthy subjects and those with no CT, along with an interaction and a dose-dependent effect of CT and CHR status on PEA levels. Such findings seemed to be independent of cannabis use [65].

### 3.8 Effect of *CNR1* and Cannabis use Over Perceived Stress and Neurocognition in FEP Patients

A single study was found investigating whether FEP patients with different genetic backgrounds regulating the endocannabinoid system and cannabis use are more prone to stress and at risk of poorer cognitive function (Tables **1**-**3**). Cannabinoid receptor type 1 (CNR1) polymorphic loci were found to modulate verbal memory and attention over time as well as perceived stress. Interestingly, a significant interaction of cannabis use and CNR1 genotype was found, such that among cannabis-using patients, the effect of genotype on verbal memory baseline scores and improvement was lost, while it remained in the non-user group. Further, overall highest perceived stress levels were found among cannabis-using patients, if carrying specific CNR1 genetic variants [66].

## 4 DISCUSSION

This is the first systematic review of all studies investigating the interplay between components of the stress response and the cannabinoid system in modulating psychosis risk and outcome in humans. Previous reviews have mainly focused on the two systems independently, summarizing the role of the hypothalamic-pituitary-adrenocortical (HPA) axis [13] or the stress response system [67] as well as of exogenous cannabinoids [6, 15] or the endocannabinoid (eCB) system [20] in increasing the risk of psychosis onset [6, 13, 20] or modulating psychosis outcome [15, 20, 67]. It is noteworthy that the evidence for an alteration and its direction in either the HPA axis/stress response system or in eCB components is not always unequivocal, possibly because of substantial methodological heterogeneity across studies [8].

Evidence from this review offers some insight into the stress-vulnerability model of psychosis, indicating HPA axis anomalies in the psychosis spectrum, both at baseline [26, 32] and following experimental [27] but not lifetime [31] stress exposure, with the latter being relevant for brain [28] and dopamine [33, 34] function as well as symptom manifestation [33]. Only one study did not find evidence of HPA axis abnormalities in schizophrenia [30]. In terms of outcome, a higher risk of incident clinical psychosis as a function of SLEs was found [58]. Also, apart from a few studies not supporting an association between CT and psychosis-related measures [40, 43, 45, 51, 60], CT, as a proxy of significant stress exposure, was found to be associated with schizotypy [35] and independently increase the risk of psychosis diagnosis and symptoms [38, 41, 42, 44, 46-48, 53, 54, 57, 61, 63] as well as earlier onset [39] and poorer outcome [36]. Further, female subjects exposed to CT [59], and earlier [47] and more severe [41] CT were found to carry a greater risk of psychosis-like experiences (PLE) [47, 59] and recent-onset psychosis (ROP) [41].

Different lines of research reviewed here also corroborate the cannabinoid-hypothesis of psychosis. Direct evidence points towards a psychosis-inducing effect of THC in otherwise healthy subjects [29], which is possibly accounted for by cognitive fusion [35]. Indirect evidence suggests that cannabis use may alter cortical [33] and striatal [34] dopamine signaling, with relevance for the onset of attenuated psychotic symptoms [33], in line with the long-lasting schizophrenia dopamine-hypothesis [68]. Also, cannabis use was found to increase the risk of PLE [46, 47, 53, 57, 61, 63], psychosis diagnosis [53] including ROP [42], incident clinical psychosis [58], psychotic symptoms in bipolar disorder [40], and poorer schizophrenia outcome [36]. However, a few studies have disconfirmed these findings [43, 45, 48, 51, 60], possibly because of a higher risk mainly in the context of recent/ current use [44, 54] as well as regular and more severe use [38, 41]. Earlier psychosis onset as a function of CU was also suggested [39]. Finally, cognitive intermediate phenotypes of psychosis were found to differ depending on CNR1 genetic variation and its interaction with cannabis use [66]. The CNR1 gene encodes for the CB1 receptor, whose localization on different neuronal subpopulations specifically modulates emotional and social behavior [69-72], with potential implications for the psychosis phenotype.

However, both preclinical and clinical studies have suggested a crosstalk between the eCB system and HPA axis and stress response, urging a deep reappraisal of the inter-relationship between these two systems, especially in the context of psychosis [8, 21]. Overall, this review indicates that the HPA axis and the eCB system, both at baseline and following an exogenous perturbation (*e.g*., stress exposure, cannabis use), interact with each other in health and disease, with relevance for the pathophysiology of psychosis. The most interesting evidence from this review regarding the interplay between the two models of psychosis is discussed in the following section.

**Fig. (2):**
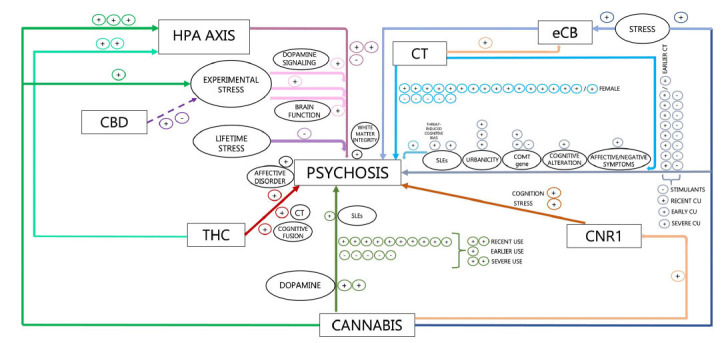
Graphical summary of the alterations in the stress response system and the endocannabinoid system reported in psychosis. **Abbreviations**: HPA, hypothalamic-pituitary-adrenal; SLEs, stressful life events; CBD, cannabidiol; CT, childhood trauma; eCB, endocannabinoid system; THC, delta-9-tetrahydrocannabinol; CU, cannabis use; COMT, catechol-O-methyltransferase gene; CNR1, cannabinoid receptor 1 gene; +, evidence of effect; -, no evidence of effect; solid line, evidence of disrupting effect; dashed line, evidence of therapeutic effect.

First, THC and CBD, two cannabis ingredients with potentially opposite effects in psychosis [73], may exert specular effects also on the HPA axis. In fact, THC seems to amplify pre-existing HPA abnormalities among psychosis patients [26] and induce transient psychotic symptoms in otherwise healthy individuals through HPA disruption [29]. Conversely, CBD is suggested to counterbalance the detrimental effects of experimental stress on the HPA axis and behavior among individuals at risk of developing psychosis [27], despite not restoring the underlying dysfunctional brain activity [28]. In line with evidence from THC studies, cannabis use was found to disrupt the HPA axis among patients at different stages of illness, including clinical high-risk (CHR) [32], ROP [31], and schizophrenia patients [30]. Interestingly, such disrupting effect of cannabis on the HPA axis was evident also among control subjects (Fig. **2**) [31].

Second, lifetime stress exposure (*e.g*., stressful life events, SLEs; childhood trauma, CT) was not found to modulate the HPA axis when the detrimental effects of cannabis use were taken into account [31]. Also, experimental stress exposure impacted patients’ HPA axis [33, 34], with relevance for the psychosis-related dopaminergic state [33, 34] and symptom severity [33], but such pattern of dopamine-cortisol response to experimental stress was reported to be blunted [33] or decoupled [34] in patients who also used cannabis (Fig. **2**).

Third, THC was found to induce more severe symptoms of psychosis in those subjects with a history of CT [35]. In line with such evidence, some [39, 40, 46, 48, 51, 53, 61, 63] but not all [36, 42-44, 49, 52, 59, 60] studies report a greater risk of PLE [46, 48, 53, 61, 63], psychotic symptoms in bipolar disorder [40], and psychosis diagnosis [51, 53] and earlier onset [39] as a function of being exposed to both cannabis use and childhood trauma (CT), with the highest risk among individuals with earlier CT [47] as well as early [45], recent [54], and regular [38] CU. Further, additional known risk factors are suggested to play a role in the interaction between CU and CT on the risk of PLE, such as the COMT gene [60, 61], white matter integrity [62], urbanicity [50, 55, 56], threat-induced cognitive biases [57], cognitive alteration [56], cognitive fusion [37], and affective comorbidity [55, 56], with SLEs being relevant for ROP [41]. Noteworthy, additional evidence supports a direct role of SLEs in conferring a higher risk of incident clinical psychosis in the context of CU (Fig. **2**) [58].

Fourth, an independent line of research indicates that either stress [64] and CT [65] affect endocannabinoid (eCB) signaling in both schizophrenia [64] and CHR [65] individuals. Also, a differential effect of cannabis use [64] as well as a dose-dependent effect of CT and psychosis risk intensification [65] on disrupting the eCB signaling have been found. Further, single evidence supports the notion that, in the context of specific CNR1 genetic vulnerability, first-episode psychosis (FEP) patients would present with higher stress and poorer memory and attention performance, with the highest perceived stress and the worst longer-term cognitive improvement in those with also a history of cannabis use (Fig. **2**) [66].

The findings of this systematic review must be seen considering some strengths and limitations. While substantially converging, evidence regarding the association between aberrancies in the stress response system and the endocannabinoid system in psychosis is limited and very heterogeneous. It is currently unclear whether the two systems affecting one other are specific to psychosis or would be commonly reported across different clinical conditions. Also, considering evidence that earlier disruption of the two biological systems may result in more severe clinical phenotypes of psychosis, their interplay needs to be further addressed by tracking illness progression in longitudinal studies. Further, potential gender-driven vulnerabilities remain to be investigated. Treatment-wise, while the therapeutic effects of CBD in psychosis seem to be reasonably mediated by its action on both systems, such line of research is still in its infancy and no other treatment strategies to restore the two systems’ function have been identified. In fact, while CBD treatment has been reported to modulate eCB signaling [74], its direct effect on CB receptors has been disconfirmed in favor of serotoninergic [75] and dopaminergic [76, 77] activities, with the modulation of the crosstalk between the dopamine and cannabinoid systems [78] being particularly relevant for CBD antipsychotic effectuates as an early treatment [79, 80].

## CONCLUSION

In conclusion, concomitant alterations in these two systems, either genetically defined (*e.g*., CNR1 genetic variation), biologically determined (*e.g*., dysfunctional HPA axis or eCB signaling), or behaviorally imputed (*e.g*., cannabis use, stress exposure, and response), are consistently reported in psychosis. Also, a complex biobehavioral perturbation is revealed not only within each system (*e.g*., cannabis use affecting the eCB tone, stress exposure affecting the HPA axis) but also across the two systems (*e.g*., THC affecting the HPA axis, CT affecting the eCB signaling) (Fig. **2**). Thus, to establish more refined biological relevance, there is a need to complementarily study the two systems’ mechanistic contribution to psychosis, rather than continuing to explore each single psychosis model.

## Figures and Tables

**Table 1:** Overview of clinical studies investigating the role of integrated hypothalamic-pituitary-adrenocortical (HPA) axis and endocannabinoid (eCB) system modulation in psychosis at different stages of illness.

**Study (Year)**	**Country**	**Aim of Study**	**Type of Study**	**Population (n)**	**Sample Size (N)**	**Outcome Measure (Test Name or Description)**
D'Souza *et al.* (2005)	United States	To assess the effect of THC on cortisol and prolactin levels in SCZ/SAD patients	1. *In vivo* cannabinoid exposure in humans 2. Quantitative HPA assessment in humans	1. HC (*n* = 22); 2. SCZ (*n* = 13): (a) PLB; (b) THC	35	HPA axis functioning (serum cortisol, serum prolactin)
Monterrubio *et al.* (2006)	Australia	To assess the association between FA, AA, and stress in SCZ-CU and SCZ-NCU patients	Quantitative eCB precursors/related lipids assessment in humans	1. SCZ-CU (*n* = 6); 2. SCZ-NCU (*n* = 6)	12	FA, AA blood levels (erythrocytes extraction)
Cougnard *et al.* (2007)	The Netherlands/ Germany	To assess the interaction and/or cumulative effect of CT, CU, and T0 PLEs over psychotic symptoms in the general population	Association between risk factors and outcomes in humans	1. General population first cohort (*n* = 4786): (a) noPLEs (*n* = 4018); (b) PLEs (*n* = 768) 2. General population second cohort (*n* = 2452): (a) noPLEs (*n* = 2223); (b) PLEs (*n* = 229)	7238	Psychosis measures (1. (a) T0, (b) T2: CIDI; 2. (a) T0: SCL-90-R; (b) T2: DIA-X/M-CIDI)
De Pradier *et al.* (2009)	France	To assess the interaction effect of CT, CU, and 5-HTTLPR polymorphism over lifetime psychotic symptoms in BPAD patients	Association between risk factors and outcomes in humans	1. Symptoms (*n* = 74) 2. No symptoms (*n* = 63)	137	Psychosis measures (DIGS, DSM-IV)
Houston *et al.* (2009)	United States	To assess the interaction of CT and early CU over psychosis diagnosis in the general population	Association between risk factors and outcomes in humans	General population cohort	5877	Psychosis measures (CIDI, SCID, DSM-III-R)
Harley *et al.* (2010)	Ireland	To assess the interaction and/or cumulative effect of CT and CU over psychotic symptoms in the general population	Association between risk factors and outcomes in humans	1. NCU+NCT (*n* = 175) 2. CU (*n* = 12); 3. CT (*n* = 18) 4. CU+CT (*n* = 6)	211	Psychosis measures (K-SADS)
Daly *et al.* (2011a)	United Kingdom	To assess the interaction effect of CT and CU over psychotic symptoms in the general population	Association between risk factors and outcomes in humans	General population cohort	3649	Psychosis measures (reported hallucinations)
Houston *et al.* (2011) Daly *et al.* (2011b)	United Kingdom	To assess the interaction effect of CT and CU over psychosis diagnosis in the general population	Association between risk factors and outcomes in humans	General population cohort	7403	Psychosis measures (phase 1: clinical interviews, PSQ; phase 2: SCAN, ICD-10 criteria)
Kuepper *et al.* (2011)	Germany	To assess the interaction effect of CT and CU over psychotic symptoms in the general population	Association between risk factors and outcomes in humans	1. NCU+NCT (*n* = 1264) 2. CU (*n* = 296); 3. CT (*n* = 266); 4. CU+CT (*n* = 97)	1923	Psychosis measures (DIA-X/M-CIDI)
Konings *et al.* (2012)	Greece/The Netherlands	1. To assess the interaction effect of early CT and later CU over psychosis risk in the general population; 2. To assess the mediating effect of early CT in the association between later CU and psychosis in the general population	Association between risk factors and outcomes in humans	1. General population first cohort: (a) NCT+NCU (*n* = 481); (b) CU (*n* = 19); (c) CT (*n* = 1059); (d) CT+CU (*n* = 77); 2. General population second cohort: (a) NCT+NCU (*n* = 3017); (b) CU (*n* = 217); (c) CT (*n* = 1363); (d) CT+CU (*n* = 245)	6478	Psychosis measures (1. CAPE; 2. CIDI psychosis G section)
Alemany *et al.* (2013)	Spain	To assess the moderating effect of COMT gene polymorphism in the interaction of CT and CU over PLEs in HS	Association between risk factors and outcomes in humans	COMT Val158Met genotypes: 1. Val/Val (*n* = 127) 2. Val/Met (*n* = 201) 3. Met/Met (*n* = 91)	533	Psychosis measures (CAPE)
Monteleone *et al.* (2013)	Italy	To assess the effect of CU on cortisol levels in chronic SCZ patients	Quantitative HPA assessment in humans	1. HC (*n* = 15) 2. SCZ-CU (*n* = 16) 3. SCZ-NCU (*n* = 12)	43	HPA axis functioning (salivary baseline cortisol, salivary CAR)
Murphy *et al.* (2013)	United States	To assess the interaction effect of CT and CU over psychosis risk in the general population	Association between risk factors and outcomes in humans	General population cohort	2355	Psychosis measures (CIDI)
van Nierop *et al.* (2013)	The Netherlands	To assess the mediating effect of CU on the link between CT and PLEs/psychotic disorder in the general population	Association between risk factors and outcomes in humans	General population cohort	6646	Psychosis measures (CIDI 20-question add-on psychosis instrument, SCID-I)
Vinkers *et al.* (2013)	The Netherlands/Belgium	To assess the interaction effect of CT, CU, and COMT gene polymorphisms over PLEs in HS	Association between risk factors and outcomes in humans	1. First study group (*n* = 918): COMT Val158Met genotypes: (a) Val/Val (20%); (b) Val/Met (50%); (c) Met/Met (30%); 2. Second study group (*n* = 339): COMT Val158Met genotypes: (a) Val/Val (24%); (b) Val/Met (49%); (c) Met/Met (27%)	1257	Psychosis measures (CAPE)
DeRosse *et al.* (2014)	United States	To assess the cumulative effect of CT, CU, PLEs, low IQ, and low PSES over WM integrity in HS	Association between risk factors and outcomes in humans	HS cohort	112	WM integrity measures (DTI, TBSS)
Mizrahi *et al.* (2014)	Canada	To assess the effect of CU and stress exposure on cortisol and dopamine brain release in CHR patients	1. *In vivo* stress exposure in humans 2. Quantitative HPA assessment in humans	1. CHR-CU (*n* = 12); 2. CHR-NCU (*n* = 12)	24	1. Stress-induced brain DA response (PET scan); 2. HPA axis functioning (salivary cortisol); 3. Stress-induced PLEs (SOPS); 4. Psychometric stress measures (SAQ)
Morgan *et al.* (2014)	United Kingdom	To assess the interaction effect of CT and CU over PLEs in the general population	Association between risk factors and outcomes in humans	General population cohort	1680	Psychosis measures (PSQ)
Barrigòn *et al.* (2015)	Spain	To assess the interaction effect of CT and CU in ROP patients	Association between risk factors and outcomes in humans	1. ROP (*n* = 60); 2. Healthy siblings (*n* = 60)	120	Psychosis measures (SCID-I, PANSS)
Guloksuz *et al.* (2015)	Germany	To assess the cumulative effect of CT and CU in the association between PE and non-psychotic disorders in the general population	Association between risk factors and outcomes in humans	General population cohort	3021	Psychosis measures (SCL-90-R)
Sideli *et al.* (2015)	United Kingdom	To assess the interaction effect of CT and CU over psychosis risk in FEP patients	Association between risk factors and outcomes in humans	1. FEP (*n* = 231): (a) NCU+NCT (*n* = 58); (b) CU (*n* = 108); (c) CT (*n* = 12); (d) CU+CT (*n* = 53); 2. HC (*n* = 214): (a) NCU+NCT (*n* = 76); (b) CU (*n* = 105); (c) CT (*n* = 14); (d) CU+CT (*n* = 19)	445	Psychosis measures (OPCRIT checklist)
Baudin *et al.* (2016)	France	To assess the interaction and/or cumulative effect of CT and CU in SCZ/SAD patients	Association between risk factors and outcomes in humans	FACE-SCZ network	366	1. Psychosis measures (SCID-1, PANSS, C-SSRS); 2. Psychosocial measures (GAF, MARS, S-QoL-18)
Carol *et al.* (2017)	United States	To assess the effect of CU on cortisol levels in CHR patients	Quantitative HPA assessment in humans	1. HC (*n* = 29); 2. CHR-CU (*n* = 17); 3. CHR-NCU (*n* = 26)	75	HPA axis functioning (salivary cortisol)
Lu *et al.* (2017)	Canada/United States	To assess the interaction effect of CT and CU over perceptual abnormalities in CHR patients	Association between risk factors and outcomes in humans	CHR cohort	441	Psychosis measures (CAPS)
Arranz *et al.* (2018)	Spain	To assess the interaction and/ or cumulative effect of CT, SLEs, and CU in ROP patients	Association between risk factors and outcomes in humans	1. HC (*n* = 61); 2. ROP (*n* = 146)	207	Psychosis measures (OPCRIT checklist v.4.0)
Pries *et al.* (2018)	The Netherlands	To assess the cumulative effect of CT and CU over PLEs in the general population	Association between risk factors and outcomes in humans	General population cohort	6646	Psychosis measures (CIDI 1.1 for PLEs, CIDI 3.0 for affective dysregulation, interviewer observation for negative symptoms, WISC-III for cognitive alteration)
Rojnic Kuzman *et al.* (2019)	Croatia	To assess the interaction effect of *CNR1* and CU over perceived stress and neurocognition in FEP patients	Association between risk factors and outcomes in humans	1. CNR1 C>T: (a) CCgen (*n* = 26); (b) CTgen (*n* = 55); (c) TTgen (*n* = 31); 2. CNR1 A>G: (a) AAgen (*n* = 102); (b) AGgen (*n* = 19)	121	1. Neurocognitive measures (MMSE, RAVLT, Digit Span F-B, BD, FAB, CDT, STROOP 1-2-3, TMTB, TMTA, Digit Symbol, ROCF, Language semantic and phonetic fluency tests); 2. Psychometric stress measures (HRSS)
Schifani *et al.* (2019)	Canada	To assess the effect of CU and stress exposure on cortisol and dopamine brain release in CHR patients	1. *In vivo* stress exposure in humans 2. Quantitative HPA assessment in humans	1. HC (*n* = 11); 2. CHR-CU (*n* = 8); 3. CHR-NCU (*n* = 14)	33	1. Stress-induced brain DA response (PET scan); 2. HPA axis functioning (salivary cortisol); 3. Stress-induced APS (SOPS); 4. Psychometric stress measures (SAQ)
Appiah-Kusi *et al.* (2020a) (United Kingdom)	United Kingdom	To assess the effect of CBD and stress exposure on cortisol levels in CHR patients	1. *In vivo* cannabinoid exposure in humans 2. *In vivo* stress exposure in humans 3. Quantitative HPA assessment in humans	1. HC (*n* = 26); 2. CHR+CBD (*n* = 16); 3. CHR+PLB (*n* = 16)	58	1. HPA axis functioning (serum cortisol); 2. Psychometric stress measures (STAI-S, SSDPS-N)
Appiah-Kusi *et al.* (2020b)	United Kingdom	To assess the effect of CT on eCBs/AEs levels in CHR patients	Quantitative eCBs/AEs assessment in humans	1. HC (*n* = 58); 2. CHR (*n* = 33)	91	eCBs/AEs plasma levels (LC-MS)
Colizzi *et al.* (2020)	United Kingdom	To assess the effect of THC on PLS and cortisol levels in HS	1. *In vivo* cannabinoid exposure in humans 2. Quantitative HPA assessment in humans	1. PLS (*n* = 11), 2. noPLS (*n* = 5): (a) PLB; (b) THC	16	HPA axis functioning (serum cortisol)
Frydecka *et al.* (2020)	Poland	To assess the mediating effect of CU and cognitive biases on the link between CT and PLEs in the general population	Association between risk factors and outcomes in humans	General population cohort	6772	Psychosis measures (PQ-16)
Labad *et al.* (2020)	Spain	To assess the effect of CT, SLEs, and CU on cortisol levels in ROP patients	Quantitative HPA assessment in humans	1. HC-CU (*n* = 8); 2. HC-NCU (*n* = 39); 3. ROP-CU (*n* = 11); 4. ROP-NCU (*n* = 45)	103	HPA axis functioning (salivary CAR, diurnal salivary cortisol slope, DSTR)
Newman-Taylor *et al.* (2020a)	United Kingdom	To assess the link between CT and THC-induced PLS in CU subjects	1. *In vivo* cannabinoid exposure in humans 2. Association between risk factors and outcomes in humans	CU cohort	20	Psychosis measures (PSI, PANSS)
Newman-Taylor *et al.* (2020b)	United Kingdom	1. To assess the link between CT and CU-induced paranoia, PLEs, and distress in CU subjects 2. To assess the link between CT and CU-induced paranoia, PLEs, and distress in psychotic patients	Association between risk factors and outcomes in humans	1. First study group (*n* = 172): CU cohort; 2. Second study group (*n* = 60): (a) CU (*n* = 38); (b) NCU (*n* = 22)	232	Psychosis measures (PS, PQB)
Carlyle *et al.* (2021)	Australia	1. To assess the moderating effect of CT on the link between CU and PLEs in CU subjects 2. To assess the mediating effect of cannabis-induced dysphoria/paranoia on the link between CT and PLEs in CU subjects	Association between risk factors and outcomes in humans	CU cohort	2630	Psychosis measures (CAPE-15)
Davies *et al.* (2021)	United Kingdom	To assess the effect of CBD and stress exposure on cortisol-brain coupling alterations in CHR patients	1. *In vivo* cannabinoid exposure in humans; 2. *In vivo* stress exposure in humans; 3. Quantitative HPA assessment in humans	1. HC (*n* = 19); 2. CHR+PLB (*n* = 17); 3. CHR+CBD (*n* = 16)	52	1. HPA axis functioning (serum cortisol); 2. Mediotemporal function (fearful face-processing fMRI)
Kirli *et al.* (2021)	Turkey	To assess the cumulative effect of SLEs, CU, and heavy drinking over psychosis risk in the general population	Association between risk factors and outcomes in humans	General population cohort	2142	Psychosis measures (phase 1: clinical interviews, CIDI; phase 2: SCID)
Lemvigh *et al.* (2021)	Denmark	To assess the interaction effect of CT, CU, and SCZ-PGRS over SCZ vulnerability and SCZ diagnosis in co-twins SCZ patients	Association between risk factors and outcomes in humans	1. Unaffected co-twins (*n* = 54): (a) MZ (*n* = 28); (b) DZ (*n* = 26); 2. Patients (*n* = 64): (a) MZ (*n* = 38); (b) DZ (*n* = 26); 3. HC (*n* = 98): (a) MZ (*n* = 58); (b) DZ (*n* = 40)	216	Psychosis measures (SCAN)
del Re *et al.* (2022)	Greece/United States	To assess the mediating effect of hippocampus and SCZ-PGRS on the interaction of CT and CU over AgePsyOnset in psychotic patients	Association between risk factors and outcomes in humans	1. HC (*n* = 397); 2. PRO (*n* = 788): (a) BPAD (*n* = 209); (b) SAD (*n* = 279); (c) SCZ (*n* = 300)	1185	Psychosis measures (AgePsyOnset, PANSS, BACS)

**Abbreviations:** AA, Arachidonic Acid; 5-HTTLPR, Functional polymorphism within the promoter region of the 5-HTT gene; AAgen, AA genotype carriers; AEs, Acylethanolamines; AgePsyOnset, Age of Psychosis Onset; AGgen, AG genotype carriers; APS, Attenuated Positive Symptoms; BACS, The Brief Assessment of Cognition in Schizophrenia; BD, Block Design test; BPAD, Bipolar Affective Disorder; CAPE, Community Assessment of Psychic Experiences; CAPS, The Content of Attenuated Positive Symptoms; CAR, Cortisol Awakening Response; CBD, Cannabidiol; CCgen, CC genotype carriers; CDT, Clock Drawing Test; CHR, Clinical High-Risk for psychosis; CIDI, Composite International Diagnostic Interview; CNR1, Cannabinoid Receptor 1 gene; CNR1 A > G, rs12720071 (CNR1 polymorphism); CNR1 C > T, rs7766029 (CNR1 polymorphism); COMT, catechol-O-methyltransferase; C-SSRS, Columbia Suicide Severity Rating Scale; CT, Childhood Trauma; CTgen, CT genotype carriers; CTQ-SF, The Childhood Trauma Questionnaire-Short Form; CU, Cannabis Use/Cannabis Users; DA, Dopamine; DIA-X/M-CIDI, Munich CIDI; Digit Span F-B, Digit span test Forwards and Backwards; DIGS, Diagnostic Interview for Genetic Study; DSM-III-R, Diagnostic and Statistical Manual for Mental Disorders, Third Edition Revised; DSM-IV, Diagnostic and Statistical Manual for Mental Disorders, Fourth Edition; DSTR, Dexamethasone Suppression Test Ratio; DTI, Diffusion Tensor Imaging; DZ, Dizygotic; eCB/eCBs, Endocannabinoid/Endocannabinoids; FA, Fatty Acid; FAB, Frontal Assessment Battery; FACE-SCZ, FondaMental Advanced Centers of Expertise in Schizophrenia; FEP, First-Episode Psychosis; fMRI, functional Magnetic Resonance Imaging; GAF, Global Assessment of Functioning; HC, Healthy Controls; HPA, Hypothalamic-Pituitary-Adrenal; HRSS, Holmes and Rahe Stress Scale, The Social Readjustment Rating Scale; HS, Healthy Subjects; IQ, Intelligence Quotient; K-SADS, Schedule for Affective Disorders and Schizophrenia for School-Age Children; LC-MS, Liquid Chromatography–Mass Spectrometry; MARS, Medication Adherence Rating Scale; MMSE, Mini Mental Status Examination; MZ, Monozygotic; n, subgroup numerosity; N, sample size; NCT, No Childhood Trauma; NCU, No Cannabis Use/Non Cannabis Users; OPCRIT, Operational Criteria Checklist for Psychotic Illness and Affective Illness; PANSS, The Positive and Negative Symptoms Scale; PET, Positron Emission Tomography; PLB, Placebo; PLEs, Psychotic-Like Experiences; PLS, Psychotomimetic symptoms; PQ-16, Prodromal Questionnaire; PQB, Prodromal Questionnaire-Brief Version; PS, Paranoia Scale; PSES, Parental Socio-Economic Status; PSI, The Psychotomimetic State Inventory; PSQ, Psychosis Screening Questionnaire; RAVLT, Rey Auditory Verbal Learning Test; ROCF, Rey-Osterrieth Complex Figure test; ROP, Recent-Onset Psychosis; SAD, Schizoaffective Disorder; SAQ, State Anxiety Questionnaire; SCAN, Schedule for Assessment in Neuropsychiatry; SCID, Structured Clinical Interview for Disorders; SCL-90-R, Self-Report Symptom Checklist-90-R; SCZ, Schizophrenia; SCZ-PGRS, Schizophrenia Polygenic Risk Score; SLEs; Stressful Life Events; SOPS, Scale of Prodromal Symptoms; S-QoL-18, Short-Quality of Life-18; SSDPS-N, Self-Statements during Public Speaking Scale; STAI-S, The State-Trait Anxiety Inventory; STROOP 1, Stroop words; STROOP 2, Stroop colors; STROOP 3, Stroop word-colors; T0, Timepoint 0; T2, Timepoint 2; TBSS, Tract-Based Spatial Statistics;THC, Delta-9-tetrahydrocannabinol; TMTA, Trail Making Test A; TMTB, Trail Making Test B; TTgen, TT genotype carriers; Val158Met, Valine(158)Methionine polymorphism; WISC-III, Wechsler Intelligence Scale for Children, Third Edition; WM, White Matter.

**Table 2:** Summary of evidence of clinical studies investigating the role of integrated hypothalamic-pituitary-adrenocortical (HPA) axis and endocannabinoid (eCB) system modulation in psychosis at different stages of illness.

**Study (Year)**	**Summary of Evidence**
D'Souza *et al.* (2005)	1. **Cortisol levels:** (a) **dose: PLB < THC**; (b) **dose x time**: 10 min: PLB *vs.* THC (all dosages), NS; **80 min**, **140 min after THC infusion: PLB < THC(all dosages)**; (c) **group x time**; (d) group x dose x time, NS; 2. **Prolactin levels:** (a) **dose: PLB < THC**; (b) **dose x time: PLB < THC**; (c) **baseline: SCZ *vs.* HC**; (d) **group x time**; (e) group x dose x time, NS
Monterrubio *et al.* (2006)	**Association between AA, FAs, and stress: SCZ-CU: ↓ AA, ↑ stress; ↓ 16,18m + sFAs, ↑ stress; ↑ LA, ↑ stress; SCZ-NCU: ↑ 16,18m + sFAs, ↑ stress**; AA, stress: NS; LA, stress: NS
Cougnard *et al.* (2007)	**Risk of psychotic symptoms at T2**: (a) **PLEs > noPLEs (EDSP sample: only for ≥ 1 risk factor)**; (b) **PLEs+CT+CU+urbanicity > noPLEs+CT +CU+urbanicity** (highest risk difference); (c) **PLEs x CT x CU x urbanicity interaction, ↑**
De Pradier *et al.* (2009)	**Risk of lifetime psychotic symptoms**: **CU x CT interaction, ↑**; **CU, ↑**; CT, NS; **5-HTT genotype (number of s alleles), ↑**; CU x genotype (number of s alleles) interaction, NS; CT x 5-HTT genotype (number of s alleles) interaction, NS; **Risk of lifetime CU or dependence**: CT, NS; genotype (number of s alleles), NS; **CT x 5-HTT genotype (number of s alleles) interaction, ↑**
Houston *et al.* (2009)	**Psychosis diagnosis**: CT, NS; CU, NS; **CT x CU interaction; CT x early CU (<16yo) interaction;** CT x later CU (>16yo) interaction, NS
Harley *et al.* (2010)	**Risk of psychotic symptoms (OR):** NCU+NCT < CT, CU; **CT+CU > NCU+NCT**; [(CU+CT) - CU - CT + (NCU+NCT)] > 0
Daly *et al.* (2011a)	**Risk of hallucinations in adulthood**: **CT (unwanted sex under 16 yo), ↑ (F)**; CT (unwanted sex under 16 yo), NS (F) (after adjustment for initial psychotic symptoms); CT (unwanted sex under 16 yo), NS (M); CU x CT (unwanted sex under 16 yo) interaction, NS (F and M)
Houston *et al.* (2011) Daly *et al.* (2011b)	**Psychosis diagnosis**: CU, NS; CT (any), NS; **CU x CT (unwanted sex under 16 yo) interaction, ↑**; early CU, NS; CT (cumulative sex trauma under 16 yo), NS; early CU x CT (cumulative sex trauma under 16 yo) interaction, NS Psychosis diagnosis: CU x CT (unwanted sex under 16 yo) interaction, NS (after adjustment for CT under 16 yo x stimulant use interaction); Risk of psychosis: CT+CU < CT+CU+stimulants
Kuepper *et al.* (2011)	Risk of positive psychotic symptoms (T2-T3 interval): CU, NS; CT, NS; CU x CT interaction, NS
Konings *et al.* (2012)	1. General population first cohort: **Main effects and early CT x later CU interaction on subsequent psychosis; ↑ CU-induced psychosis, ↑ early CT**; 2. General population second cohort: (a) **Main effects and early CT x CU interaction on subsequent psychosis;** (b) **CU risk and frequency: Early CT > Non-CT**
Alemany *et al.* (2013)	1. Effect on positive PLEs: CU, NS; CT, NS (after adjustment); COMT, NS; CU x CT interaction, NS; CU x COMT interaction, NS; CT x COMT interaction, NS; **CU x CT x COMT interaction**; 2. Effect on negative PLEs: CU, NS (after adjustment); CT, NS (after adjustment); COMT, NS; CU x CT interaction, NS; CU x COMT interaction, NS; CT x COMT interaction, NS; CU x CT x COMT interaction, NS
Monteleone *et al.* (2013)	1. **Baseline cortisol levels: SCZ-CU > HC**; SCZ-NCU *vs.* HC, NS; SCZ-CU continued after onset *vs.* SCZ-CU stopped after onset, NS; PANSS scores, NS 2. **CAR-Delta max: SCZ-CU < HC**; SCZ-NCU *vs.* HC, NS; SCZ-CU continued after onset *vs.* SCZ-CU stopped after onset, NS; PANSS scores, NS
Murphy *et al.* (2013)	1. **CT at 16, CT<16yo: F > M**; **CU: M > F**; Pre-CT CU: F *vs.* M, NS; **Post-CT CU: F > M**; **Visual+Auditory hallucinations: F > M**; other comparisons, NS; 2. Pre-CT psychopathology and psychosis prevalence: **ADHD, SAD, SP, psychosis: F > M; CD, IED: M > F**; other comparisons, NS; 3. **Adult psychosis experiences: pre-CT psychosis, SP, childhood rape, ↑;** CU, NS; **CT x CU interaction, ↑**; other comparisons, NS
van Nierop *et al.* (2013)	Psychotic experience: **↑ CT, ↑** psychotic experience; **↑ CT, ↑** psychotic experience severity; **↑ CT, ↑** psychotic disorder; **↑ CU, ↑** psychotic experience severity; (PLEs, SCZ/schizophreniform disorder diagnosis): CT → (CU) → psychotic experiences, NS (mediation)
Vinkers *et al.* (2013)	1. **Effects on PLEs (first study group)**: **↑ CT, ↑ PLEs; ↑ CU, ↑ PLEs**; COMT, NS; CT x COMT interaction, NS; **CU x COMT interaction, ↑ PLEs**; **CT x CU interaction, ↑ PLEs**; **CT x CU x COMT interaction, ↑ PLEs**; 2. Effects on PLEs (second study group): **↑ CT, ↑ PLEs;** CU, NS; **COMT, ↑ PLEs**; **CT x COMT interaction, ↑;** CU x COMT interaction, NS; CT x CU interaction, NS; CT x CU x COMT interaction, NS
DeRosse *et al.* (2014)	Effect on Fractional Anisotropy: **↑ CR (CT, CU, PLEs, IQ, PSES), ↓ Fractional Anisotropy in the SLF**; **↑ PLEs, ↓ Fractional Anisotropy in the SLF**; **↓ PSES, ↓ Fractional Anisotropy in the SLF**; other single risk factors, NS
Mizrahi *et al.* (2014)	1. **Task performance** (**number of errors/timeouts**): **CHR-CU (MIST) > CHR-CU (SMCT)**; **CHR-NCU (MIST) > CHR-NCU (SMCT)**; other comparisons, NS; 2. **Subjective stress respose:** (a) **MIST > SMCT (all groups)**; (b) **strain**, (c) **tense**, (d) **upset: CHR-CU > CHR-NCU**; (e) **satisfied: CHR-CU < CHR-NCU**; 3. **Stress-induced PLEs:** (a) **positive SOPS: CHR-CU (MIST) > CHR-CU (screening); CHR-NCU (MIST) > CHR-NCU (screening)**; CHR-CU (SMCT) *vs.* CHR-CU (screening), NS; CHR-NCU (SMCT) *vs.* CHR-NCU (screening), NS; (b) **negative SOPS: CHR-CU (screening) < CHR-NCU (screening)**; (c) **APS (following MIST): CHR-NCU (pre-scan) < CHR-NCU (post-scan)**; **CHR-CU (pre-scan) < CHR-CU (post-scan)**; (d) **APS (following SMCT): CHR-NCU (pre-scan) < CHR-NCU (post-scan)**; CHR-CU (post-scan) *vs.* CHR-CU (pre-scan), NS; 4. **Stress-induced brain DA response (BPND)**: (a) **wholeSTR**, (b) **SMST: CHR-NCU (MIST) < CHR-NCU (SMCT); CHR-CU (MIST) > CHR-CU (SMCT); CHR-CU > CHR-NCU**; (c) **LST:** CHR-NCU (MIST) < CHR-NCU (SMCT) (trend effect); **CHR-CU (MIST) > CHR-CU (SMCT); CHR-CU > CHR-NCU**; (d) **SN**: CHR-NCU (MIST) < CHR-NCU (SMCT) (trend effect); CHR-CU (MIST) *vs.* CHR-CU (SMCT), NS; **CHR-CU > CHR-NCU**; (e) GP: CHR-NCU (MIST) *vs.* CHR-NCU (SMCT), NS; **CHR-CU (MIST) > CHR-CU (SMCT);** CHR-CU > CHR-NCU (trend effect); (f) **AST: CHR-NCU (MIST) < CHR-NCU (SMCT); CHR-CU (MIST) > CHR-CU (SMCT);** CHR-CU *vs.* CHR-NCU, NS**;** greater %change BPND: earlier cannabis use onset (trend effect); 5. Stress-induced salivary cortisol (AUC): (a) CHR-NCU *vs.* CHR-CU, NS; (b) %change MIST *vs.* SMCT: positive association with BPND in AST and wholeSTR (CHR-NCU only)
Morgan *et al.* (2014)	Synergistic effects of CT and CU over **PLEs: ↑ CT, ↑ PLEs (OR)**; lifetime CU, NS; **↑ past year CU, ↑ PLEs (OR)**; CT x lifetime CU interaction (ICR), NS; **CT x past year CU interaction, ↑ PLEs (ICR; trend effect)**
Barrigòn *et al.* (2015)	**Risk of developing psychosis (OR): CU, ↑ (after adjustment)**; **CT, ↑ (after adjustment)**; **neuroticism, ↑ (after adjustment)**; CT x CU interaction, NS; other interactions, NS
Guloksuz *et al.* (2015)	1. **Cumulative effect size (OR) of PE and EE (CU, CT, urbanicity) on affective spectrum disorder: unexposed < PE, EE, PE+EE, 2EE, PE+2EE, 3EE, PE+3EE**; 2. PE+3EE: highest cumulative OR
Sideli *et al.* (2015)	Risk of FEP: CU, NS; CT, NS; **CU x CT interaction (OR),** ↑; CU x CT interaction (ICR), NS; low potency CU, NS; **high potency CU, ↑**; less than daily CU, NS; **daily CU, ↑**
Baudin *et al.* (2016)	1. **↑ number of hospitalizations, ↑ PANSS total, ↑ PANSS positive, ↑ PANSS excitement, ↑ PANSS emotional distress, ↓ GAF, ↓ S-QoL-18: ** **↑ CT**, other associations NS 2. **↓ age of onset, ↓ age at first hospitalization, ↓ MARS: ↑ CU**, other associations NS 3. **CT x CU interaction on SCZ/SAD course:** ↑ age of onset; ↓ age at first hospitalization; ↑ number of hospitalizations; **↑ total duration of hospitalizations**; 4. CT x CU interaction on SCZ/SAD clinical characteristics: ↑ PANSS total; ↑ PANSS positive; ↑ PANSS negative; ↑ PANSS disorganized; ↑ PANSS excitement; ↑ PANSS emotional distress; ↑ number of suicide attempts per year of illness; 5. CT x CU interaction on SCZ/SAD psychosocial characteristics: ↓ GAF; ↑ MARS; ↓ S-QoL-18
Carol *et al.* (2017)	1. **Salivary cortisol levels (AUCg, past week cannabis use): HC < CHR-NCU, CHR-CU**; CHR-NCU *vs.* CHR-CU, NS; **↑ use, ↑ disorganized symptoms** (other symptoms, NS) 2. **Salivary cortisol levels (AUCg, past month cannabis use): CHR-CU > HC**; CHR-CU > CHR-NCU (trend effect); CHR-NCU *vs.* HC, NS; ↑ frequency of use, ↑ cortisol (trend effect); symptoms, NS
Lu *et al.* (2017)	Logistic regression model predicting **simple auditory and visual perceptual abnormalities**: **↑ CT, ↑ abnormalities**; past CU, NS; **↑ current CU, ** **↑ abnormalities**; CT x CU interaction, NS
Arranz *et al.* (2018)	**Environmental factors interaction and cumulative effect on psychosis**: 1. **↑ CT, ↑ ROP risk**; 2. **↑ CU, ↑ ROP risk**; 3. **Combined effect of CT, SLEs, and CU: significant association with psychosis only for ≥ 2 risk factors**; 4. Exposed to all factors: highest ROP risk
Pries *et al.* (2018)	**Dose–response relationship between the risk-loading (CT, CU, family history of affective disorders, urbanicity, foreign born, hearing impairment) and PLEs (OR): No RF < 1 RF < 2 RF < more than 2 RF**; Risk loading → (Affective dysregulation; negative symptoms; cognitive alteration) → ↑ PLEs (moderation)
Rojnic Kuzman *et al.* (2019)	1. **Neurocognitive improvement over 18 months**: (a) **CNR1 C>T (rs7766029): CCgen ↑ executive functions, verbal memory, attention** (adjusted only for baseline neurocognition); **CCgen ↑ verbal memory** (executive functions NS, attention NS; adjusted for baseline neurocognition, gender, age, negative symptoms, and CU); **genotype x CU interaction on verbal memory;** (b) **CNR1 rs12720071: AGgen ↑ executive functions, ↓ language functions than AAgen** (adjusted only for baseline neurocognition); AGgen *vs.* AAgen, NS (adjusted for baseline neurocognition, gender, age, negative symptoms, and CU); **genotype x negative symptoms x CU interaction on BD**; 2. **Perceived stress change:** (a) **rs7766029 and rs12720071** (adjusted only for baseline neurocognition); **rs7766029,** rs12720071, NS (adjusted for baseline neurocognition, gender, age, negative symptoms, and CU); (b) **CU x both genotypes interaction**
Schifani *et al.* (2019)	1. **Subjective stress response:** (a) **SMCT: HC < CHR-CU, CHR-NCU**; CHR-CU *vs.* CHR-NCU, NS; (b) **MIST: CHR-NCU > HC**; other comparisons, NS; 2. **Stress-induced DA response in PFC** (**ΔBPND**): (a) dlPFC: all comparisons, NS; (b) **mPFC: CHR-CU < CHR-NCU**; other comparisons, NS; positive association with ΔAUC1 in mPFC, dlPFC; negative association with APS in mPFC, dlPFC; 3. **Stress-induced salivary cortisol** (**ΔAUC1**): **CHR-NCU > CHR-CU,** HC (trend effect); CHR-CU *vs.* HC, NS; **↑ ΔAUC1, ↑ ΔBPND** 4. **Stress-induced APS**: CHR-NCU (pre-stress) *vs.* CHR-NCU (post-stress), NS; **CHR-CU (post-stress) > CHR-CU (pre-stress), CHR-NCU (post-stress); ↑ post-stress APS, ↓ ΔBPND**
Appiah-Kusi *et al.* (2020a)	1. **Stress-induced serum cortisol change (10 min after TSST minus baseline)**: **HC > CHR+PLB, CHR+CBD**; CHR+CBD > CHR+PLB; 2. **Stress-induced anxiety reaction** (**STAI-S AUC**): **CHR+PLB > HC**; CHR+PLB > CHR+CBD; CHR+CBD *vs.* HC, NS; 3. **Effect of stress on negative self-statements** (**SSDPS-N AUC**): **CHR+PLB > HC**; CHR+PLB > CHR+CBD (trend effect); CHR+CBD *vs.* HC, NS
Appiah-Kusi *et al.* (2020b)	1. **Group differences on AEs/eCBs plasma levels**: (a) **OEA**, (b) **AEA**, (c) **2-AG: CHR > HC**; (d) PEA: CHR > HC (trend effect); 2. (a) **CT effect: ↑ PEA, AEA, 2-AG levels**; (b) **CHR effect: ↑ AEA, 2-AG levels**; (c) **CHRxCT effect: ↑ PEA levels**; ↑ AEA levels (trend effect); 3. **Effects of 2 *vs.* 1 RF on AEs/eCBs plasma levels**: (a) **OEA**, (b) **AEA**, (c) **PEA**, (d) **2-AG: 2RF > 1RF**; 4. **Effects of RF number on AEs/eCBs plasma levels**: (a) **OEA**, (b) **AEA**, (c) **PEA**, (d) **2-AG: noRF < 1RF < 2RF**; 5. **↑ PEA levels: ↑ total CAARMS score; ↑ total CTQ score**; 6. ↑ AEA levels: ↑ total CAARMS score (trend effect)
Colizzi *et al.* (2020)	1. **Serum cortisol change (baseline minus 2.5 h post-drug injection)**: **PLB > THC**; 2. **THC-induced serum cortisol change (THC change minus PLB change): PLS < noPLS**
Frydecka *et al.* (2020)	**Effects on PLEs (APS): ↑ CU, ↑ PLEs; ↑ CT, ↑ PLEs;** CT → (CU) → (Threat-induced cognitive biases) → ↑ PLEs (mediation)
Labad *et al.* (2020)	1. **Diurnal salivary cortisol change: ROP-CU < ROP-NCU; HC-CU < HC-NCU**; ROP-CU *vs.* HC-CU, NS; SLEs, NS; CT, NS 2. Salivary CAR, 3. DSTR: all comparisons, NS
Newman-Taylor *et al.* (2020a)	Inter-correlations for study variables: **↑ CT, ↑ Schizotypy (O-LIFE)**; CT, PSI (T1): NS; CT, PSI (T2): NS; **↑ CT, ↑ PSI (change)**; CT, PSI (change): NS (after adjustment for cognitive fusion); CT, PANSS (T1): NS; **↑ CT, ↑ PANSS (T2)**; **↑ CT, ↑ PANSS (change)**; CT, PANSS (change): NS (after adjustment for cognitive fusion)
Newman-Taylor *et al.* (2020b)	1. First study group: (a) **Prediction of CU-related symptoms** (MANOVA): **↑ CT, ↑ paranoia**; **↑ CT, ↑ PLEs**; **↑ CT, ↑ distress**; (b) **Prediction of CU-related symptoms** (MANCOVA): **↑ CT, ↑ paranoia (external attribution and cognitive fusion as covariates)**; CT, PLEs: NS (external attribution and cognitive fusion as covariates); **↑ CT, ↑ distress (external attribution as covariate)**; CT, distress: NS (cognitive fusion as covariate); 2. Second study group: (a) **Prediction of CU-related symptoms** (MANOVA): **↑ CT, ↑ paranoia**; **↑ CT, ↑ PLEs**; CT, distress: NS; (b) **Prediction of CU-related symptoms** (MANCOVA): CT, paranoia: NS (external attribution as covariate); CT, PLEs: NS (external attribution as covariate); **↑ CT, ↑ paranoia (cognitive fusion as covariate)**; **↑ CT, ↑ PLEs (cognitive fusion as covariate)**; CT, distress: NS
Carlyle *et al.* (2021)	**Effects on PLEs (frequency): ↑ CU, ↑ PLEs; ↑ CT, ↑ PLEs**; **CU → (CT) → ↑ PLEs (moderation)**; **CT x CU interaction, ↑ PLEs; CT → (CU-induced dysphoria/paranoia) → ↑ PLEs (mediation)**
Davies *et al.* (2021)	1. **Cortisol and fMRI response to fear processing post-TSST:** (a) **HC group: ↓ right parahippocampal activation, ↑ cortisol;** (b) **HC *vs.* CHR+PLB**; (c) CHR+PLB *vs.* CHR+CBD, NS; 2. Anxiety and fMRI response post-TSST: (a) HC group: right parahippocampal activation, anxiety, NS; (b) HC *vs.* CHR+PLB, NS; (c) CHR+PLB *vs.* CHR+CBD, NS
Kirli *et al.* (2021)	Incident clinical psychosis (OR): **CU use and frequency, ↑**; **SLEs exposure (at least 3 events) and number, ↑**; Combined effect of SLEs, CU, and heavy drinking: higher association with psychosis for ≥ 2 risk factors
Lemvigh *et al.* (2021)	1. Logistic regression model predicting **illness vulnerability (PRO *vs.* HC)**: **↑ CT, ↑ vulnerability**; **↑ SCZ-PGRS, ↑ vulnerability**; sporadic CU, NS; **regular CU, ↑ vulnerability**; CT x sporadic CU interaction, NS; **CT x regular CU interaction, ↑**; 2. Logistic regression model predicting **illness status (Patients *vs.* Unaffected co-twins)**: **↑ regular CU, ↑ status**; other predictors, NS
del Re *et al.* (2022)	1. **AgePsyOnset:** (a) **CU < NCU**; (b) **↑ CT, ↓ AgePsyOnset** (both in CU and NCU); **↑ CU, ↓ AgePsyOnset** (both in high CT and low CT); (c) **CT x CU interaction, ↓ AgePsyOnset**; **survival differs significantly for various CU levels at the different CT levels**; (d) **Total CT, ↓ AgePsyOnset**; **Direct CT, ↓ AgePsyOnset**; **CT → (CU) → ↓ AgePsyOnset (mediation)**; **CT → (HP) → ↓ AgePsyOnset (mediation)** 2. **Positive PANSS score**: **↑ CTQ score, ↑**; **↓ AgePsyOnset, ↑**; **↓ LaHP, RaHP, LpHP volume, ↑**; RpHP volume, NS; **↑ BACS, ↓**; 3. Negative PANSS score: CTQ score, NS; AgePsyOnset, NS; HP volume (all areas), NS; **↑ BACS, ↓**; 4. Relationship between variables: **↓ HP, ↓ BACS (whole PRO sample, PRO with CU before AgePsyOnset)**; NS in PRO with CU after AgePsyOnset; 5. Variables **predicting PRO group**: **CU, higher CT, low total BACS, small LpHC, higher SCZ-PGRS score**; other variables, NS

**Note**: ≥, At least; <, Smaller than; >, Greater than; ↑, Increased; ↓, Decreased; 16.18 m + sFAs, 16- and 18-carbon monounsaturated and saturated FAs; 2-AG, 2-Arachidonoylglycerol; → (x) →, Mediation/Moderation effect; **Abbreviations:** AA, Arachidonic Acid; AAgen, AA genotype carriers; ADHD, Attention-Deficit Hyperactivity Disorder; AEA, Anandamide; AEs, Acylethanolamines; AgePsyOnset, Age of Psychosis Onset; AGgen, AG genotype; carriers; aHP, Anterior hippocampus; APS, Attenuated Positive Symptoms; AST, Associative striatum; AUC, Area Under the Curve; BACS, The Brief Assessment of Cognition in Schizophrenia; BPND, Binding Potential relative to Nondisplaceable Binding; CAARMS, The Comprehensive Assessment of At Risk Mental States; CAR, Cortisol Awakening Response; CBD, Cannabidiol; CCgen, CC genotype carriers; CD, Conduct Disorder; CHR, Clinical High-Risk for psychosis; COMT, catechol-O-methyltransferase; CR, Cumulative Risk); CT, Childhood Trauma; CU, Cannabis Use/Cannabis Users; DA, Dopamine; dlPFC, dorsolateral PFC; DSTR, Dexamethasone Suppression Test Ratio; eCB/eCBs, Endocannabinoid/Endocannabinoids; EE, Environmental Exposure; F, Female; FA, Fatty Acid; FEP, First-Episode Psychosis; fMRI, functional Magnetic Resonance Imaging; GAF, Global Assessment of Functioning; GP, Globus pallidus; h, hours; HC, Healthy Controls; ICR, Interaction Contrast Ratio; IED, Intermittent Explosive Disorder; IQ, Intelligence Quotient; L, Left; LA, Linoleic Acid; LST, Limbic striatum; M, Male; MANCOVA, Multivariate Analysis of Covariance; MANOVA, Multivariate Analysis of Variance; MARS, Medication Adherence Rating Scale; min, minutes; MIST, The Montreal Imaging Stress Task; mPFC, medium PFC; NCT, No Childhood Trauma; NCU, No Cannabis Use/Non Cannabis Users; NS, Not Significant; OEA, Oleoylethanolamide; O-LIFE, The Oxford-Liverpool Inventory of Feelings and Experiences; OR, Odd Ratio; PANSS, The Positive and Negative Symptoms Scale; PE, Psychosis Expression; PEA, Palmitoylethanolamide; PFC, Prefrontal cortex; pHP, Posterior hippocampus; PLB, Placebo; PLEs, Psychotic-Like Experiences; PLS, Psychotomimetic symptoms; PRO, Probands; PSES, Parental Socio-Economic Status; PSI, The Psychotomimetic State Inventory; R, Right; RF, Risk Factor; ROP, Recent-Onset Psychosis; SAD, Schizoaffective Disorder; SCZ, Schizophrenia; SCZ-PGRS, Schizophrenia Polygenic Risk Score; SLEs, Stressful Life Events; SLF, Superior Longitudinal Fasciculus; SMCT, Sensorimotor Control Task; SMST, Sensorimotor striatum; SN, Substantia nigra; SOPS, Scale of Prodromal Symptoms; SP, Specific Phobia; S-QoL-18, Short-Quality of Life-18; SSDPS-N, Self-Statements during Public Speaking Scale; STAI-S, The State-Trait Anxiety Inventory; T2, Timepoint 2; T3, Timepoint 3; THC, Delta-9-tetrahydrocannabinol; TSST, Trier Social Stress Test; *vs.*, *versus*; wholeSTR, Whole striatum; yo, years old; ΔAUC, Variation in Area Under the Curve; ΔBPND, Variation in Binding Potential relative to Nondisplaceable Binding. Bold font emphasizes statistically significant results.

**Table 3:** Methodological quality of clinical studies investigating the role of integrated hypothalamic-pituitary-adrenocortical (HPA) axis and endocannabinoid (eCB) system modulation in psychosis at different stages of illness.

**Study (Year)**	**Study Design**	**Defined Study Population**	**Age (Years)**	**Female %**	**HPA Axis/** **Stress Measure**	**Adequate HPA Axis/Stress ** **Evaluation**	**eCB System Measure**	**Adequate eCB System ** **Evaluation**	**Control Group**	**Comparability of Subjects**	**Excluded/Adjusted for Confounding Factors**	**Statistical ** **Analyses**	**F/S**
D'Souza *et al*. (2005)	√ Analytic, observational, interventional	√ SCZ/SAD patients: DSM-IV; CU ≥ once, without lifetime CUD	√ 1. HC: 29 ± 11.6; 2. SCZ: 44.46 ± 10.4	√ 1. HC: 8 (36.36%); 2. SCZ: 3 (23.08%)	√ 1. Serum cortisol; 2. Serum prolactin	√ Multiple assessment (baseline 60 min prior to THC, 10 min, 80 min, 140, 200 min after THC)	√ THC 2.5 or 5 mg (iv administration)	√ Single administration (3 test days)	√ HC; SCZ + PLB	√/X Matched for lifetime CU; not matched for age, education, SES, tobacco use	√ Excluded if clinical instability (recent or current hospitalization, homicidality, suicidality, and/or severe disability), cannabis-naive individuals, recent abuse (3 months), or dependence (1 year) on substances (excluding tobacco)	√ Normal probability plots, Kolmogorov-Smirnov test, variance-covariance matrix according to the AIC, pairwise comparisons, Bonferroni's correction, nonparametric approach with group as a between-subjects factor, relative effects plots	√
Monterrubio *et al*. (2006)	√ Analytic, observational	√ CZP-treated SCZ patients	√ 1. SCZ-CU: 29-48; 2. SCZ-NCU: 25-56	√ 1. SCZ-CU: 17%; 2. SCZ-NCU: 50%	√ Perceived stress	√ Single assessment (DASS, 21-question-version)	√ Lifetime CU	√ Single assessment (semi-structured interview; CU if ≥ 4 t/m for ≥ 6 months)	√ SCZ-NCU	√/X Matched for age, gender, disorder severity, diet, depression, anxiety, and stress; not matched for CZP dosage	√ Excluded if IQ < 70, neurological disorder, lipid disorder, SUD (other than cannabis); Adjusted for CZP dosage	√ ANCOVA	√
Cougnard *et al*. (2007)	√ Descriptive, qualitative, analytic, observational	√ 1. NEMESIS sample; 2. EDSP sample	√ 1. NEMESIS sample: 41.02 ± 11.9; 2. EDSP sample: 18.3 ± 3.3	√ 1. NEMESIS sample: 2559 (53%); 2. EDSP sample: 1196 (48.8%)	√ CT (1. NEMESIS sample: sexual and/or physical and/or emotional and/or psychological abuse; 2. EDSP sample: sexual abuse, physical threat, serious accident)	√ Single assessment (at T0: 1. NEMESIS sample: semi-structured interview; 2. EDSP sample: M-CIDI, PTSD module)	√ Lifetime CU	√ Single assessment (at T0: 1. NEMESIS sample: CIDI-L CU section; EDSP sample: DIA-X/M-CIDI)	X	NA	√ Adjusted for age, gender, education, any T0 CIDI lifetime DSM-III-R diagnosis	√ Additive interaction analysis (risk difference regression analysis)	√
De Pradier *et al*. (2009)	√ Descriptive, qualitative, analytic, observational	√ BPAD patients: DIGS, DSM-IV based	√ 1. Symptoms: 44.5 ± 14.6 (22-78); 2. No symptoms: 55.7 ± 6.33 (25-74)	√ 1. Symptoms: 67% (49/74); 2. No symptoms: 62% (39/63)	√ CT (sexual abuse)	√ Single assessment (TQH)	√ Lifetime CU or cannabis dependence	√ Single assessment (DIGS, DSM-IV based)	√ No symptoms	√/X Matched for gender, lifetime alcohol, and drug use or dependence, CT; not matched for lifetime CU or dependence, 5-HTTLPR genotype	√ Excluded if lack of fluency in French, SAD diagnosis	√ Chi-square test, t-test Armitage test, logistic regression analysis	√
Houston *et al*. (2009)	√ Descriptive, qualitative, analytic, observational	√ NCS sample	√ 32.02 ± 10.59 (15-54)	√ 51.9%	√ CT (sexual abuse)	√ Single assessment (CIDI, PTSD module)	√ Lifetime CU	√ Single assessment (CIDI, Medication and Drug module)	√ NCT; NCU	NA	√ Adjusted for age, gender, ethnicity, education, urbanicity, employment status, depression, living situation	√ Hierarchical logistic regression analysis	X
Harley *et al*. (2010)	√ Descriptive, qualitative, analytic, observational	√ Adolescents "at-risk": SDQ (clinical range); CDI (clinical range and/or "I want to kill myself" on item 9)	√ 12-15	X	√ CT (sexual and/or physical abuse, domestic violence)	√ Single assessment (K-SADS)	√ Lifetime CU	√ Single assessment (K-SADS)	√ NCU + NCT; CU; CT	√ Matched for gender, education	√ Adjusted for age, gender, SES, family psychiatric history	√ Logistic regression analysis	√
Daly *et al*. (2011a)	√ Descriptive, qualitative, analytic, observational	√ 1970 British Birth Cohort sample	X	X	√ CT (sexual abuse, non-consensual sex before 16 yo)	√/X Single assessment (self-report)	√ CU prior to 29 yo	√/X Single assessment (self-report)	X	NA	√ Adjusted for SES, initial psychotic symptoms	√ Logistic regression analysis	X
Houston *et al*. (2011) Daly *et al*. (2011b)	√ Descriptive, qualitative, analytic, observational	√ AMPS sample	√ 51.12 ± 18.59;	√ 3801 (51.34%)	√ CT (sexual abuse, non-consensual sex before 16 yo)	√ Single assessment (SCAN - Domestic Violence and Abuse section)	√ Lifetime CU	√ Single assessment (SCAN - Drugs section)	X	NA	√ Adjusted for CT after 16 yo, age, gender, education, ethnicity, employment, depression, alcohol √ Adjusted for age, gender, CT under 16 yo x stimulant use interaction	√ Chi-square test, binary logistic regression analysis	X
Kuepper *et al*. (2011)	√ Descriptive, qualitative, analytic, observational	√ EDSP sample	√ T0: 18.3 ± 3.3; T2: 21.8 ± 3.4; T3: 26.6 ± 3.5	√ 51.8%	√ Lifetime trauma (war, sexual and/or physical abuse, rape, natural disasters, accidents, kidnapping, hostage-taking, witnessing any of the previous)	√ Single assessment (DIA-X/M-CIDI, at T0)	√ 1. Lifetime CU; 2. Interval CU (T0-T2)	√ 1. and 2. Double assessment (DIA-X/M-CIDI, at T0, T2: defined if ≥ 5 times)	√ NCU + NCT; CU; CT	NA	√ Excluded if lifetime psychotic experiences at T2; Adjusted for age, gender, SES, baseline CU, baseline other drug use, urbanicity	√ Logistic regression analysis	X
Konings *et al*. (2012)	√ Descriptive, qualitative, analytic, observational	√ 1. Greek national perinatal study; 2. NEMESIS sample	√ 1. Greek national perinatal study: 7-19; 2. NEMESIS: 18-64	√ 1. Greek national perinatal study: 55%; 2. NEMESIS: 53%	√ CT (sexual and/or physical and/or emotional abuse)	√ Single assessment (Greek national perinatal study: parental questionnaire; NEMESIS: semi-structured interview)	√ Lifetime CU	√ Single assessment (Greek national perinatal study: CAPE, additional questions; NEMESIS: CIDI-L CU section)	√ NCU + NCT; CU; CT	NA	√ Adjusted for gender, urbanicity, other drug use, age, ethnicity, relationship status, discrimination experience, unemployment	√ Wald test, Logistic regression analysis	√
Alemany *et al*. (2013)	√ Descriptive, qualitative, analytic, observational	√ HS: ADV in university offices and schools	√ 22.9 ± 5.4	√ 54.6%	√ CT (sexual and/or physical and/or emotional abuse)	√ Single assessment (CTQ)	√ Lifetime CU	√/X Single assessment (single-item question about the frequency of consumption: ‘never’, ‘once’, ‘monthly’, ‘weekly’ or ‘daily’)	X	NA	√ Excluded if major medical illness affecting brain function, neurological disorder, current SU, history of head trauma, history of psychiatric treatment; Adjusted for age, gender, schizotypy (SPQ-B), trait anxiety (STAI)	√ Multiple linear regression analysis, logistic regression analysis, log-likelihood ratio test, Bonferroni’s correction	√
Monteleone *et al*. (2013)	√ Analytic, observational	√ Chronic SCZ patients: DSM-IV TR; ≥ 3-month stable antipsychotic treatment	√ 1. HC: 37.6 ± 6.9; 2. SCZ-CU: 39.1 ± 7.2; 3. SCZ-NCU: 43.6 ± 7.3	√ 1. HC: 20.00%; 2. SCZ-CU: 12.50%; 3. SCZ-NCU: 50.00%	√ Salivary cortisol	√ Multiple assessment (CAR, 15 min, 30 min, 60 min after awakening)	√ CU prior to SCZ onset	√ Single assessment (clinical records, SCID-IP, questionnaire)	√ HC; SCZ-NCU	√ Matched for age, gender	√ Excluded if a history of head trauma or endocrinological disorder (DM2 included), ongoing hormonal therapies	√ ANOVA, Tukey's test, Chi-square test, Pearson's correlation test	√
Murphy *et al*. (2013)	√ Descriptive, analytic, observational	√ NCS-R sample	√ 44.34 ± 17.27	√ 57.8%	√ CT (sexual abuse)	√ Single assessment (CIDI 3.0, PTSD module)	√ Lifetime CU	√ Single assessment (CIDI, SU module)	√ NCT; NCU	NA	√ Adjusted for age, gender, education, income, pre-trauma pathology, sexual traumatic experiences >16 yo	√ Hierarchical regression analysis	X
van Nierop *et al*. (2013)	√ Descriptive, qualitative, analytic, observational	√ NEMESIS-2 sample	√ 44	√ 55%	√ CT (sexual and/or physical and/or emotional abuse, physical and/or emotional neglect)	√ Single assessment (questionnaire based on the NEMESIS-1 trauma questionnaire)	√ Lifetime CU	√ Single assessment (CIDI 3.0, Illegal SU section)	X	NA	√ Excluded if insufficient fluency in Dutch; Adjusted for age, gender	√ Logistic regression analysis, linear regression analysis, mediation analysis	√
Vinkers *et al*. (2013)	√ Descriptive, qualitative, analytic, observational	√ 1. HS from Discovery sample (Cannabis Quest): 18-25 yo; Dutch-speaking; 2. HS from Replication sample (GROUP study): 16-50 yo; Dutch-speaking	√ 1. Discovery sample: 20 (18-40); 2. Replication sample: 32 (16-56)	√ 1. Discovery sample: 53%; 2. Replication sample: 57%	√ CT (sexual and/or physical and/or emotional abuse, physical and/or emotional neglect)	√ Single assessment (CTQ)	√ 1. Discovery sample: current CU; 2. Replication sample: past year CU	√ Single assessment (1. Discovery sample: ≥ 3 euros/week during the past month or longer; 2. Replication sample: CIDI)	X	NA	√ 1. Discovery sample: excluded if a personal history of psychiatric disorder (SCID, MINI); 2. Replication sample: excluded if a personal history of psychotic disorder, family history of psychotic disorder in first- or second-degree relatives	√ MANCOVA, logistic regression analysis, interaction analysis	√
DeRosse *et al*. (2014)	√ Descriptive, qualitative, analytic, observational	√ Healthy adult volunteers	√ 36.07 ± 13.23 (18.47-68.04)	√ 46.43% (52/112)	√ CT (sexual and/or physical and/or emotional abuse, physical and/or emotional neglect)	√ Single assessment (CTQ)	√ Lifetime CU	√ Single assessment (SCID-I/ NP, more than once before 18 yo)	X	√ Matched for age, gender, and ethnicity across the range of CR	√ Excluded if the first-degree relative with psychotic disorder, current or lifetime psychotic disorder, current or lifetime affective disorder, active or recent substance abuse, contraindications to MRI, previous psychosurgery, pregnancy; Adjusted for age, gender	√ Linear regression analysis (permutation-based test), TCFE (for inference on the statistic maps), correlation analysis, ANCOVA, stepwise multiple regression analysis	√
Mizrahi *et al*. (2014)	√ Analytic, observational, interventional	√ CHR individuals: COPS criteria (SIPS/SOPS); “moderately ill” on CGI or < 52 on GAF or > 9 on SOPS-P	√ 1. CHR-CU: 24.25 ± 4.7; 2. CHR-NCU: 23.00 ± 4.6	√ 1. CHR-CU: 50.00%; 2. CHR-NCU: 41.67%	√ 1. Salivary cortisol; 2. Stress-induced anxiety	√ 1. Multiple assessment (every 12 min through scan); 2. Single assessment (SAQ after MIST task)	√ Lifetime or current CU or CD	√ Multiple assessment (MCQ, ≥ 3 t/w CU; DSM-IV criteria for CD; UDS)	√ CHR-NCU	√/X Matched for age, gender, mother PBI, education; not matched for tobacco use	√ Excluded if current or lifetime axis I psychotic disorder, current or lifetime > 4-week antipsychotic treatment, past or current CNS disorder; CHR-NCU excluded if past 6 months substance abuse or SD	√ ANOVA, Linear regression analysis	√
Morgan *et al*. (2014)	√ Descriptive, qualitative, analytic, observational	√ SELCoH sample	√ 16-29: 572 participants (28.7%); above 30: 1108 (71.2%)	√ 66.6% (946)	√ 1. CT (sexual and/or physical abuse); 2. SLEs	√/X Single assessment (single-item question about physical abuse; single-item question about sexual abuse; nine more questions for SLEs)	√ 1. Lifetime CU; 2. Past year CU	√/X Single assessment (single-item question)	X	NA	√ Excluded if less than 16 yo; Adjusted for age, gender, ethnicity, education, social class	√ Logistic regression analysis	√
Barrigòn *et al*. (2015)	√ Descriptive, qualitative, analytic, observational	√ ROP patients: DSM-IV; < 5 years of illness; having a free-of-psychosis sibling	√ 1. ROP: 31.1 ± 8.1; 2. Healthy siblings: 32.3 ± 10.6	√ 1. ROP: 53% (31/58) 2. Healthy siblings: 64% (37/58)	√ CT (sexual and/or physical and/or emotional abuse, emotional neglect)	√/X Single assessment (semi-structured interview)	√ Lifetime CU	√ Single assessment (CIDI)	√ Healthy siblings	√/X Matched for age, gender, and education; not matched for marital status, temperament, CT, and CU	√ Excluded if a history of head trauma with loss of consciousness for more than 1 h, history of neurological disorder, history of somatic disorder with neurological components; Adjusted for age, gender, education, marital status, CT, CU, temperament, and differences between families	√ McNemar test, t-test, conditional logistic regression analysis	√
Guloksuz *et al*. (2015)	√ Descriptive, qualitative, analytic, observational	√ EDSP sample (adolescents, young adults)	√ T0: 18.26 ± 3.34; T1: 16.72 ± 1.19; T2: 21.74 ± 3.39; T3: 26.62 ± 3.47	√ T0: 49.26%; T1: 48.13%; T2: 49.10%; T3: 48.64%	√ 1. CT (sexual abuse and/or physical threat); 2. Interval trauma	√ Multiple assessment (DIA-X/M-CIDI at T0, T1, T2, T3)	√ 1. Lifetime CU; 2. Interval CU	√ Multiple assessment (DIA-X/M-CIDI at T0, T1, T2, T3)	X	NA	√ Adjusted for non-psychotic psychopathology load (SCL-90-R)	√ Logistic regression analysis, interaction analysis	√
Sideli *et al*. (2015)	√ Descriptive, qualitative, analytic, observational	√ FEP patients: ICD-10 criteria (F20-F29; F30-F34)	√ 1. FEP: 28.1 ± 9.1; 2. HC: 27.6 ± 9.0	√ 1. FEP: 36.8%; 2. HC: 45.8%	√ CT (sexual and/or physical abuse)	√ Single assessment (CECA.Q)	√ Lifetime CU	√ Single assessment (CEQmv)	√ NCU + NCT; CT; CU	√/X Matched for age and gender; not matched for education, ethnicity, and family psychiatric history	√ FEP excluded if an organic cause for psychosis; HC excluded if current or lifetime axis I psychotic disorder, IQ < 70, poor English fluency; Adjusted for gender, ethnicity, education, and family psychiatric history	√ Logistic regression analysis	√
Baudin *et al*. (2016)	√ Descriptive, qualitative, analytic, observational	√ SCZ/SAD patients: DSM-IV-TR	√ 32.00 ± 10.12	√ 25.14%	√ CT (sexual and/or physical and/or emotional abuse)	√ Single assessment (CTQ)	√ Lifetime CUD	√ Single assessment (DSM-IV-TR criteria)	X	NA	√ Adjusted for gender, and age (except the age of onset and age at first hospitalization analyses that were only adjusted for age)	√ Three-step multiple regression analysis, Spearman's Rho correlation test, chi-square test	√
Carol *et al*. (2017)	√ Analytic, observational	√ CHR individuals: recent moderate levels of APS (3 to 5 SIPS score); and/or past 12-month decline in global functioning with SPD; and/or past 12-month decline in global functioning with first-degree relative history of psychotic disorder	√ 1. HC: 17.34 ± 2.82; 2. CHR-CU: 19.38 ± 1.11; 3. CHR-NCU: 18.38 ± 2.01	√ 1. HC: 13M/16F; 2. CHR-CU: 16M/12F; 3. CHR-NCU: 7M/11F	√ Salivary cortisol	√ Multiple assessment (three samples collected within 2 h; every 60 min; from 8:45 AM to 2 PM)	√ 1. Past week CU; 2. Past month CU	√ Single assessment (1. UDS; 2. AUS/DUS)	√ HC; CHR-NCU	√/X Matched for gender, and education; not matched for age, anxiety, and depression	√ All participants excluded if history of head trauma, neurological disorder, lifetime substance dependence; CHR excluded if presence of axis I psychotic disorder; HC excluded if axis I disorder, first-degree relative history of psychotic disorder, THC positive UDS, self-reported lifetime CU; Adjusted for age	√ ANCOVA, t-test, chi-square test, AUCg calculation, Spearman's Rho correlation test	√
Lu *et al*.	√ Descriptive, qualitative, analytic, observational	√ CHR individuals from NAPLS-2: COPS criteria (SIPS)	√ 18.74 ± 4.17	√ 42.2% (186/441)	√ CT (sexual and/or physical and/or emotional abuse, emotional neglect)	√ Single assessment (Childhood Trauma and Abuse scale)	√ 1. Lifetime CU; 2. Current CU	√/X Single assessment (CU scale)	X	NA	√ Excluded if current axis I psychotic disorder, history of axis I psychotic disorder, IQ < 70, current CNS disorder, history of CNS disorder, current SD disorder (DSM-IV), prodromal symptoms due to axis I disorder or to drug abuse	√ Mann-Whitney U-test, Spearman correlations, chi-square test, t-test, univariate logistic regression analysis, multiple logistic regression analysis	√
Arranz *et al*. (2018)	√ Descriptive, qualitative, analytic, observational	√ ROP patients: no organic cause for psychotic symptoms	√ 1. HC: 23 (19-27); 2. ROP: 22 (18-26)	√ 1. HC: 29 (47.50%); 2. ROP: 52 (35.60%)	√ 1. CT (sexual and/or physical and/or emotional abuse); 2. Past 6 months SLEs	√ Single assessment (1. CTQ-SF; 2. HRSS)	√ Past 6 months CU	√ Single assessment (EIP-designed interview)	√ HC	√/X Matched for age, gender, ethnicity, and family history of psychotic disorders; not matched for education or tobacco use	√ All participants were excluded if pregnancy, IQ < 70, severe head injury or neurological disease, or active substance dependence (other than tobacco or cannabis); HC was excluded if a past or current history of psychiatric disorder; Adjusted for age, gender, education, ethnicity, and family history of psychosis (Effect of number of environmental factors on psychosis risk)	√ Univariate logistic analyses, hierarchical multiple regression analyses, multivariate logistic regression analyses, chi-square test	√
Pries *et al*. (2018)	√ Descriptive, qualitative, analytic, observational	√ NEMESIS-2 sample	√ 1. T0: 44.3 ± 12.5; 2. T1 (3-year follow-up): 47.6 ± 12.4; 3. T2 (6-year follow-up): 50.9 ± 12.3	√ 1. T0: 55.3% (3672/6646); 2. T1: 55.1% (2922/5303); 3. T2: 55.4% (2558/4618)	√ CT (sexual and/or physical and/or emotional abuse, emotional neglect, peer victimization)	√ Single assessment (questionnaire based on the NEMESIS-1 trauma questionnaire)	√ 1. Lifetime CU; 2. Interval CU	√ Multiple assessment (CIDI 3.0, Illegal SU section qt T0, T1, T2)	X	NA	√ Adjusted for age, gender, education	√ Logistic regression analysis	√
Rojnic Kuzman *et al*. (2019)	√ Analytic, observational	√ FEP patients: ICD-10 criteria	√ 1. CNR1 C>T: (a) CCgen: 22-30; (b) CTgen: 22-29; (c) TTgen: 21-33; 2. CNR1 A>G: (a) AAgen: 22-31; (b) AGgen: 21-29	√ 1. CNR1 C>T: (a) CCgen: 31%; (b) CTgen: 34.5%; (c) TTgen: 42%; 2. CNR1 A>G: (a) AAgen: 35%; (b) AGgen: 37%	√ Perceived stress	√ Single assessment (HRSS)	√ 1. CNR1 gene; 2. CU	√ Single assessment (1. polymorphism genotyping; 2. clinical interview for family history recollection)	√ CNR1 C>T CTgen/TTgen; CNR1 A>G AAgen	NA	√ Excluded if developmental disorder presenting with psychotic symptoms; use of medication that can provoke psychosis; addiction (other than self-reported CU); Adjusted for baseline neurocognition, gender, age, negative symptoms, CU	√ Benjamini-Hochberg method, Spearman rank correlation, ANOVA, Markov chain method, and LD test for HWE	√
Schifani *et al*. (2019)	√ Analytic, observational, interventional	√ CHR individuals: COPS criteria	√ 1. HC: 24.91 ± 5.54; 2. CHR-CU: 22.13 ± 2.80; 3. CHR-NCU: 22.07 ± 3.38	√ 1. HC: 6M/5F; 2. CHR-CU: 6M/8F; 3. CHR-NCU: 7M/11F	√ 1. Salivary cortisol; 2. Stress-induced anxiety	√ 1. Multiple assessment (every 15 min through scan; 6 samples, starting 15 min prior to injection, and 9 min prior to stress task); 2. Single assessment (SAQ after MIST task)	√ Current CU	√ 1. Single assessment (SCID-5; Semi-structured interview: cannabis use ≥ 3 t/w for ≥ 2 months or CUD) 2. Multiple assessment (UDS: positive at screening visit and on PET scan days, negative at baseline visit; CHR-CU avoiding cannabis 12 h prior to MIST)	√ HC; CHR-NCU	√ Matched for age, gender, tobacco use, PET scan parameters	√ CHR excluded if SUD except for cannabis (SCID-5), past or current antipsychotic treatment; HC excluded if past or current history of psychiatric disorder or prodromal syndrome, recreational CU or psychoactive drug use, no first-degree relative with MMD	√ GLMs, posthoc ANOVA, Bonferroni's correction, t-test, Pearson's correlation	√
Appiah-Kusi *et al*. (2020a)	√ Analytic, observational, interventional	√ CHR individuals: CAARMS criteria	√ 1. HC: 23.91 ± 3.93; 2. CHR + CBD: 22.33 ± 5.12; 3. CHR + PLB: 25.12 ± 5.40	√ 1. HC: 52.00%; 2. CHR + CBD: 37.50%; 3. CHR + PLB: 58.80%	√ 1. Serum cortisol; 2. Stress-induced anxiety; 3. Perceived stress	√ Multiple assessment (1. baseline 60 min prior to TSST, 0 min, 10 min, 20 min after TSST; 2. STAI-S post TSST; 3. SSDPS post TSST)	√ CBD 600 mg (oral administration)	√ Daily administration (7 days)	√ HC; CHR + PLB	√/X Matched for age, gender, and current CU; not matched for education	√ HC excluded if a history of MMD, positive PSQ, different geographical area	√ ANOVA, t-test, AUC	√
Appiah-Kusi *et al*. (2020b)	√ Analytic, observational	√ CHR individuals: CAARMS criteria	√ 1. HC: 25.05 ± 4.90; 2. CHR: 23.82 ± 5.28	√ 1. HC: 47.00%; 2. CHR: 49.00%	√ 1. CT; 2. Perceived stress	√ Single assessment (1. CTQ; 2. PSS)	√ 1. Current CU; 2.eCBs/AEs serum levels	√ Single assessment (1. CEQ; 2. LC-MS)	√ HC	√ Matched for age, gender, current CU	√ Excluded if a history of a previous psychotic or manic episode, past or current CNS disorder, current SD (DSM-IV), IQ < 70, any contraindications to CBD treatment or MRI, drug use during the entire study	√ ANCOVA, t-test, chi-square, correlation analysis	√
Colizzi *et al*. (2020)	√ Analytic, observational, interventional	√ HS	√ 24.44 ± 4.29	√ 9 (56.25%)	√ Serum cortisol	√ Multiple assessment (baseline; 20 min, 2.5 h after THC injection)	√ THC 1.19 mg/2 ml (iv administration)	√ Single administration	√ PLS + PLB; noPLS + PLB; noPLS + THC	NA	√ Excluded if a personal history of psychiatric illness, family history of psychiatric disorder in first-degree relatives, history of alcohol abuse, nicotine dependence, or illicit drug use	√ ANOVA, t-test, correlation analysis	√
Frydecka *et al*. (2020)	√ Descriptive, qualitative, analytic, observational	√ Polish Research Consortium online survey: CAWI method	√ 26.6 ± 4.7	√ 3433 (61.2%)	√ 1. CT: (a) emotional abuse, bullyism, emotional neglect; (b) sexual abuse, sexual harassment; 2. Threat- and safety behavior-related cognitive biases	√ Single assessment (1. TEC; 2. CECA.Q; 3. DACOBS-18)	√ Past year CU	√ Single assessment CPQ)	X	NA	√ Excluded if a personal history of past 6 months SD, personal history of psychiatric illness, personal history of neurological disease, history of antipsychotic treatment; Adjusted for age, gender, and education	√ Kolmogorov-Smirnov test, Spearman rank correlation coefficients, Mann-Whitney U test, mediation analysis	√
Labad *et al*. (2020)	√ Analytic, observational	√ ROP outpatients: psychotic disorder < 3 years, 64% FEP (SCAN)	√ 1. HC: 23.8 ± 4.5; 2. ROP: 24.8 ± 5.4	√ 1. HC: 46.80%; 2. ROP: 37.50%	√ 1. Salivary cortisol; 2. CT; 3. SLEs	√ 1. Multiple assessment (CAR (T1), 30 min (T2), 60 min (T3) after awakening, 10:00 h (T4), 23:00 h (T5), next day 10:00 h for DSTR (T6)); 2. and 3. Single assessment (CTQ-SF; HRSS)	√ Current CU	√/X Single assessment (semi-structured interview)	√ HC-NCU; ROP-NCU	√/X Matched for age and gender; not matched for tobacco use and BMI	√ HC excluded if a past or current history of psychiatric disorder	√ ANCOVA, t-test, chi-square test, multiple linear regression analysis	√
Newman-Taylor *et al*. (2020a)	√ Analytic, observational, interventional	√ CU recruited *via* electronic or paper ADV: DSM-5 (SCID); English-speaking; normal BMI	√ 19-35	√ 50%	√ CT (sexual and/or physical and/or emotional abuse, physical and/or emotional neglect)	√ Single assessment (CTQ-SF)	√ 1. THC 15 mg (oral administration); 2. Lifetime CU; 3. Current CU	√ 1. Single administration (prior to test); 2. Single assessment (single-item question about lifetime CU; UDS)	X	NA	√ Excluded if current psychiatric disorder (SCID), history of psychiatric disorder, history of psychiatric disorder in first-degree relatives, current CU more than once per week, current other drugs use, alcohol dependence, tobacco dependence, pregnancy; Adjusted for cognitive fusion	√ Shapiro-Wilk test, Pearson’s correlation, partial correlation analysis	√
Newman-Taylor *et al*. (2020b)	√ Descriptive, qualitative, analytic, observational	√ 1. First study group: past-year CU; 2. Second study group: psychotic patients from CMHSs (SCZ, FEP, SAD, post-natal psychosis, BPAD with psychotic symptoms)	√ 1. First study group: 26.45 ± 11.2 (16-75); 2. Second study group: 39.73 ± 13.06 (17-69)	√/X Second study group: 36.7% (22/60)	√ CT (sexual abuse)	√ Single assessment (ST)	√ Lifetime CU	√ Single assessment (single-item question about lifetime CU; single-item question about the age of first use; single-item question about past 3 months use; single-item question about CU as self-medication)	√/X Second study group: NCU	X	X	√ First study group: MANOVA, MANCOVA, mediation analysis; Second study group: MANOVA, MANCOVA	X
Carlyle *et al*. (2021)	√ Descriptive, qualitative, analytic, observational	√ CU youth: online university correspondence, social media ADV, SU-related websites ADV	√ 19.24 ± 2.58	√ 1332 (50.64%)	√ CT (sexual and/or physical and/or emotional abuse, physical and/or emotional neglect)	√ Single assessment (CTQ)	√ 1. Past month CU; 2. Cannabis-induced dysphoria/ paranoia	√ Single assessment (1. WHO ASSIST; 2. CEQ)	X	NA	√ Excluded if past month NCU; Adjusted for age, indigenous status, family history of SU, family history of psychiatric disorder, GAD-7, PHQ-9, CU (only for mediation analysis)	√ Moderation analysis, mediation analysis, bootstrapping test	√
Davies *et al*. (2021)	√ Analytic, observational, interventional	√ CHR individuals: CAARMS criteria	√ 1. HC: 24.30 ± 4.73; 2. CHR-CBD: 22.70 ± 5.08; 3. CHR-PLB: 24.10 ± 4.48	√ 1. HC: 10 (52.60%); 2. CHR + CBD: 10 (62.5%); 3. CHR + PLB: 7 (41.2%)	√ 1. Serum cortisol; 2. Stress-induced anxiety	√ 1. Multiple assessment (baseline 60 min prior to TSST, 0 min, 10 min, 20 min after TSST); 2. Single assessment (STAI-S post-TSST)	√ 1. CBD 600 mg (oral administration); 2. CBD plasma levels	√ 1. Daily administration (7 days); 2. Multiple assessment (baseline 60 min prior to TSST, 0 min, 10 min, and 20 min after TSST)	√ HC; CHR + PLB	√/X Matched for age, gender, ethnicity, UDS results, current CU, and current tobacco use; not matched for education	√ Excluded if a history of previous psychotic or manic episode, current SD (DSM-IV) (except cannabis), IQ < 70, CNS disorder or severe intercurrent illness, any contraindications to CBD treatment or MRI	√ Linear regression analysis, t-test, chi-square test	√
Kirli *et al*. (2021)	√ Descriptive, qualitative, analytic, observational	√ Random sample	√ 15-64	√ 1278 (59.6%)	√ SLEs	√ Double assessment (List of Threatening Life Events Questionnaire)	√ 1. Lifetime CU; 2. Interval CU	√ Double assessment (defined if ≥ 5 times)	X	NA	√ Excluded if the presence of psychosis at baseline; Adjusted for age, gender, education, and social insurance	√ Cramer’s V index, logistic regression analysis	√
Lemvigh *et al*. (2021)	√ Descriptive, qualitative, analytic, observational	√ Co-twins SCZ patients	√ 1. HC: 40.64 ± 10.20; 2. Patients: 41.09 ± 10.53; 3. Unaffected co-twins: 40.37 ± 10.51	√ 1. HC: 49.0% (48/98); 2. Patients: 45.3% (29/64); 3. Unaffected co-twins: 51.9% (28/54)	√ CT (sexual and/or physical and/or emotional abuse, physical and/or emotional neglect)	√ Single assessment (CTQ)	√ Lifetime CU	√/X Single assessment (single-item question about the frequency of consumption: ‘never’, ‘a few times’, ‘regular consumption’; single-item question about methods of use)	√ HC; Unaffected co-twins	√ Matched for age, gender	√ All participants were excluded if there was a history of head trauma, diagnosis of alcohol/drugs addiction, presence of serious clinical illness, or pregnancy; HC was excluded if psychosis was diagnosed in first-degree family members	√ t-test, chi-square test, Wilcoxon-signed rank test, logistic regression analysis, Bonferroni’s correction	√
del Re *et al*. (2022)	√ Descriptive, qualitative, analytic, observational	√ Psychotic patients (PRO group - BPAD or SAD or SCZ): DSM IV-TR	√ 1. HC: 34.30 ± 11.98; 2. PRO: (a) BPAD: 36.11 ± 11.38; (b) SAD: 39.81 ± 11.72; (c) SCZ: 38.12 ± 11.91	√ 1. HC: 58.70% (233/397); 2. PRO: (a) BPAD: 56.93% (119/209); (b) SAD: 52.68% (147/279); (c) SCZ: 38.67% (116/300)	√ CT	√ Single assessment (CTQ)	√ Lifetime CU	√ Single assessment (SCID)	√ HC	√/X Not matched for age, gender, ethnicity, lifetime CU, total CT, BACS composite score, PANSS total score, PANSS subscales	√ All participants were excluded if premorbid IQ < 60, major neurological or cognitive disorder, serious medical, neuro-opthalmological, or neurological illness affecting CNS, pregnancy, breastfeeding, positive pregnancy test on MRI day, contraindications to MRI, recent SUD, persistent drug dependence, positive UDS; HC was excluded if a personal history of psychosis, BPAD, or recurrent MDD, family history of SCZ, SAD, or BPAD in first-degree relatives; Adjusted for age, gender, ethnicity, MRI acquisition site, and eTIV (in addition for hippocampus)	√ ANCOVA, chi-square test, MANOVA, regression analysis, Kaplan-Meyer survival analysis, Log-rank (Mantel-Cox) testing	√

**Abbreviations**: ≥, At least; 5-HTTLPR, Functional polymorphism within the promoter region of the 5-HTT gene; AAgen, AA genotype carriers; ADV, advertising; AEs, Acylethanolamines; AGgen, AG genotype carriers; AIC, Akaike's Information Criterion; AM, Ante Meridiem; AMPS, Adult Psychiatric Morbidity Survey; ANCOVA, Analysis of covariance; ANOVA, Analysis of variance; APS, Attenuated Positive Symptoms; ASSIST, Alcohol, Smoking and Substance Involvement Screening Test; AUC, Area Under the Curve; AUS/DUS, Alcohol/Drug Use Scale; BACS, The Brief Assessment of Cognition in Schizophrenia; BMI, Body Mass Index; BPAD, Bipolar Affective Disorder; CAARMS, The Comprehensive Assessment of At Risk Mental States; CAPE, Community Assessment of Psychic Experiences; CAR, Cortisol Awakening Response; CAWI, Computer Assisted Web Interview; CBD, Cannabidiol; CCgen, CC genotype carriers; CDI, Children's Depression Inventory; CECA.Q, Childhood Experience of Care and Abuse Questionnaire; CEQmv, Cannabis Experiences Questionnaire modified version; CGI, Clinical Global Impression; CHR, Clinical High-Risk for psychosis; CIDI, Composite International Diagnostic Interview; CMHSs, Community Mental Health Services; CNR1, Cannabinoid Receptor 1 gene; CNR1 A>G, rs12720071 (CNR1 polymorphism); CNR1 C>T, rs7766029 (CNR1 polymorphism); CNS, Central Nervous System; COPS, Criteria of Prodromal Syndromes; CR, Cumulative Risk; CT, Childhood Trauma; CTgen, CT genotype; carriers; CTQ, The Childhood Trauma Questionnaire; CTQ-SF, The Childhood Trauma Questionnaire—Short Form; CU, Cannabis Use/Cannabis Users; CUD, Cannabis Use Disorder; CZP, Clozapine; DACOBS-18, Davos Assessment of Cognitive Biases Scale; DASS, Depression Anxiety and Stress Scales; DIA-X/M-CIDI, Munich CIDI; DIGS, Diagnostic Interview for Genetic Study; DM2, Type 2 Diabetes; DSM-III-R, Diagnostic and Statistical Manual for Mental Disorders, Third Edition Revised; DSM-IV, Diagnostic and Statistical Manual for Mental Disorders, Fourth Edition; DSM-IV-TR, Diagnostic and Statistical Manual for Mental Disorders, Fourth Edition, Text Revision; DSM-5, Diagnostic and Statistical Manual for Mental Disorders, Fifth Edition; DSTR, Dexamethasone Suppression Test Ratio; eCB/eCBs, Endocannabinoid/Endocannabinoids; EDSP, The German Early Developmental Stages of Psychopathology; EIP, Early Psychosis Program; eTIV, estimated Total Intracranial Volume; F, Female; F/S, Description of Funding and/or Sponsorship; F20-29 or F30-34, Satisfied criteria for psychosis; FEP, First-Episode Psychosis; GAD-7, Generalized Anxiety Disorder-7; GAF, Global Assessment of Functioning; GLMs, Generalized linear models; GROUP, Genetic Risk and Outcome of Psychosis; h, hours; HC, Healthy Controls; HPA, Hypothalamic-Pituitary-Adrenal; HRSS, Holmes and Rahe Stress Scale, The Social Readjustment Rating Scale; HS, Healthy Subjects; HWE, Hardy-Weinberg equilibrium; ICD-10, International Classification of Diseases, 10th Revision; IQ, Intelligence Quotient; iv, Intravenous; K-SADS, Schedule for Affective Disorders and Schizophrenia for School-Age Children; LC-MS, Liquid Chromatography-Mass Spectrometry; LD, Linkage disequlibrium; M, Male; MANCOVA, Multivariate Analysis of Covariance; MANOVA, Multivariate Analysis of Variance; MCQ, Marijuana Craving Questionnaire; MDD, Major Depressive Disorder; mg, Milligrams; min, minutes; MINI, The Mini International Neuropsychiatric Interview; MIST, The Montreal Imaging Stress Task; ml, Milliliters; MMD, Major Mental Disorder; MRI, Magnetic Resonance Imaging; NA, Not Applicable; NAPLS, North American Prodrome Longitudinal Study; NCS, National Comorbidity Survey; NCS-R, National Comorbidity Survey-Replication; NCT, No Childhood Trauma; NCU, No Cannabis Use/Non Cannabis Users; NEMESIS, The Netherlands Mental Health Survey and Incidence Study; NP, Non-Patient edition of SCID; PANSS, The Positive and Negative Symptoms Scale; PBI, Parental Bonding Index; PET, Positron Emission Tomography; PHQ-9, Patient Health Questionnaire-9; PLB, Placebo; PLS, Psychotomimetic symptoms; PM, Post Meridiem; PRO, Probands; PSS, Perceived Stress Scale; PSES, Parental Socio-Economic Status; PTSD, Post-Traumatic Stress Disorder; ROP, Recent-Onset Psychosis; SAD, Schizoaffective Disorder; SAQ, State Anxiety Questionnaire; SCAN, Schedules for Clinical Assessment in Neuropsychiatry; SCID, Structured Clinical Interview for Disorders; SCL-90-R, Self-Report Symptom Checklist-90-Revised; SCZ, Schizophrenia; SD, Substance Dependence; SDQ, Strengths and Difficulties Questionnaire; SELCoH, The South East London Community Health Study; SES, Socio-economic status; SIPS, Structured Interview for Prodromal Syndromes; SLEs, Stressful Life Events; SOPS, Scale of Prodromal Symptoms; SPD, Schizotypal Personality Disorder; SPQ-B, Schizotypy Personality Questionnaire-Brief; ST, The Childhood Sexual Trauma Questionnaire; STAI-S, The State-Trait Anxiety Inventory; SU, Substance Use; SUD, Substance Use Disorder; t/m, times per month; T0, Timepoint 0; T1, Timepoint 1; T2, Timepoint 2; T3, Timepoint 3; T4, Timepoint 4; T5, Timepoint 5; T6, Timepoint 6; TCFE, Threshold-Free Cluster Enhancement; TEC, Traumatic Events Checklist; THC, Delta-9-tetrahydrocannabinol; TQH, Trauma Questionnaire History; TSST, Trier Social Stress Test; TTgen, TT genotype carriers; UDS, Urine Drug Screening; yo, years old; WHO, World Health Organization.

## LIST OF ABBREVIATIONS

AEs: Acylethanolamines
CAR: Cortisol Awakening Response
CB1: Cannabinoid Receptor Type 1
CHR: Clinical High-risk
CSF: Cerebrospinal Fluid
CT: Childhood Trauma
eCB: Endocannabinoid
HPA: Hypothalamic-pituitary-adrenocortical
PLE: Psychosis-like Experiences

## STANDARDS OF REPORTING

PRISMA guidelines and methodology were followed.

## SUPPLEMENTARY MATERIAL

PRISMA checklist is available as supplementary material on the publisher’s website along with the published article.
